# Human hippocampal ripples tune cortical responses based on predicted uncertainty

**DOI:** 10.1038/s41593-026-02345-6

**Published:** 2026-06-25

**Authors:** Darya Frank, Stephan Moratti, Robin Hellerstedt, Johannes Sarnthein, Ningfei Li, Andreas Horn, Lukas Imbach, Lennart Stieglitz, Antonio Gil-Nagel, Rafael Toledano, Karl J. Friston, Bryan A. Strange

**Affiliations:** 1 Laboratory for Clinical Neuroscience, Centre for Biomedical Technology, Universidad Politécnica de Madrid, IdISSC, Madrid, Spain; 2 Andrew Mayes Centre for Cognitive Neuroscience, University of Manchester, Manchester, UK; 3 Department of Experimental Psychology, Complutense University of Madrid, Madrid, Spain; 4 Department of Neurosurgery, University Hospital and University of Zurich, Zurich, Switzerland; 5 Neuroscience Center Zurich, University of Zurich and ETH Zurich, Zurich, Switzerland; 6 Movement Disorders and Neuromodulation Unit, Department of Neurology, Charité—Universitätsmedizin Berlin, corporate member of Freie Universität Berlin and Humboldt-Universität zu Berlin, Berlin, Germany; 7 Department of Neurosurgery, Harvard Medical School, Massachusetts General Hospital, Boston, MA USA; 8 Brigham & Women’s Hospital, Center for Brain Circuit Therapeutics, Boston, MA USA; 9 Swiss Epilepsy Center, Klinik Lengg, Zurich, Switzerland; 10 Epilepsy Unit, Department of Neurology, Hospital Ruber Internacional, Madrid, Spain; 11 Wellcome Trust Centre for Neuroimaging, Institute of Neurology, University College London, London, UK; 12 IDG/McGovern Institute for Brain Research, Peking University, Beijing, China

**Keywords:** Neuroscience, Cognitive neuroscience, Perception, Visual system, Hippocampus

## Abstract

To encode information efficiently, our perceptual system should detect when situations are unpredictable (that is, informative) and modulate brain dynamics to prepare for encoding. Under uncertainty, there is an increased need to generate predictions about upcoming information, a process that has been proposed to require coordinated activity between the hippocampus and neocortex. Here we show, with direct recordings from the human hippocampus and visual cortex, that after exposure to unpredictable visual stimulus streams, hippocampal ripple activity increases in frequency and duration before stimulus presentation. Prestimulus hippocampal ripples suppress changes in visual cortex gamma activity associated with uncertainty and modulate poststimulus prediction error gamma responses in higher-level visual cortex to surprising stimuli. We reveal a function of hippocampal ripples in facilitating the propagation of visual stimuli based on the expected information gain. These results, therefore, link hippocampal ripples with predictive coding accounts of neuronal message passing and precision-weighted prediction errors, revealing a mechanism relevant for perceptual synthesis and subsequent memory encoding.

## Abstract (web summary)

Using direct recordings from the human brain, Frank et al. show that hippocampal ripples increase ahead of unpredictable events and tune the visual cortex to respond faster and more strongly to surprising stimuli that follow.

## Main

An efficient recognition system should be able to prioritize the information that will constrain or inform perceptual representations^1^ and, eventually, be accumulated or retained. One way to facilitate this is to continuously generate predictions about upcoming inputs and to retain information—that is, revise beliefs—when these predictions are violated. Prediction is considered central in this account of the brain, by building a generative model of the world to minimize prediction error when sampling the sensorium^2,3^. In predictive coding formulations, predictions are transmitted in a top-down manner, and, when there is a mismatch between the predicted and the observed input, a bottom-up prediction error is returned to update or revise the source of predictions at a higher hierarchical level^4,5^. Crucially, prediction errors are weighted by the level of uncertainty (that is, their precision) associated with the given context^6^, balancing top-down and bottom-up information streams to scale the influence of prior predictions and sensory evidence, respectively^7^. This is sometimes framed in terms of precision-weighted prediction errors that instantiate the Kalman gain in Bayesian filtering formulations of predictive coding^1,4^. The implicit encoding of uncertainty lends a dual aspect to predictive processing that encompasses both the predictions of a particular sensation and predictions of its predictability (that is, precision) that modulate the influence of the ensuing prediction error^8–13^. Notably, these generative properties of predictive processing rely on ongoing integration of sensory inputs with internally generated, experience-dependent sequences, and are therefore thought to involve hippocampal–neocortical interactions^14–16^.

A hippocampal role in prediction is likely related to its function in extracting statistical regularities^17–20^ that can be applied to new situations^21,22^. Therefore, the hippocampus should represent the expected information gain of an event before it occurs—as a function of predictability^16,20^—and estimate the validity of the prediction upon observation of the event^23^. Indeed, the hippocampus has long been postulated to hold a cognitive map that is used to form predictions about upcoming inputs^24–27^. Such predictive information follow successor-like representation^28,29^, for example, in place cell firing^30^. The cortex may also have its own predictive role through communication across deep layers feeding-back predictions to the superficial layers of preceding regions in the processing hierarchy^29,30^. Mechanistically, predictions and their violation have been associated with spiking activity^31^ and oscillatory dynamics in the cortex^32,33^. Specifically, gamma-band activity has been associated with bottom-up prediction errors (reflecting surprise signals from primary sensory cortices) and alpha/beta oscillations with top-down predictions (from higher-level regions such as prefrontal cortex)^34–36^.

However, the mechanism through which predictions of future sensory inputs are generated in the hippocampus and communicated to the cortex is still unclear^16^. Irrespective of these mechanisms, they should manifest as a differential modulation of prediction–error responses in the visual cortex, depending on the predictability of the current context, which we hypothesize is itself recognized and broadcast through hippocampal processing. Specifically, when upcoming stimuli are unpredictable, they are inherently informative in the sense that they resolve uncertainty when observed (technically, they have a greater expected information gain). This leads to the hypothesis that the hippocampus has a role in precision weighting by modulating the electrophysiological correlates of prediction errors—that is, event-related gamma activity in the visual hierarchy—as a function of predictability (or entropy).

Hippocampal sharp-wave ripples (SWRs; ripples henceforth) are found in every investigated mammalian brain and consist of sharp waves (large-amplitude, negative-polarity activity resulting from synchrony in the apical dendritic layer of CA1 pyramidal neurons) and ripples (~140 to 200 s^−1^ in rodents, resulting from interactions between excitatory and inhibitory neurons in CA3 (refs. ^37,38^)). They are viewed as a preconscious mechanism to explore the organism’s options, searching for past experiences to extrapolate and predict future outcomes^27,39^. This is in addition to their role in replay of past experiences^38,40^. As prospection relies on past experiences and is mediated by the hippocampus^25^, ripples may also underlie anticipation of future outcomes to guide subsequent behavior. Indeed, ripples have been shown to portend behavior in the immediate future, in the form of experienced trajectories^41^ and new paths^42,43^, as well as preplay of future events during sleep^44^. Specifically, the sequential firing pattern that occurs during rodent SWRs, in addition to ‘replay’ of spatial trajectories, has been shown to reflect all physically available trajectories within the environment not realized in prior behavior^43^, and trajectories taken by participants in subsequent goal-directed navigation^42^. Furthermore, there is evidence that cortical activity is modulated in a peri-ripple manner, both through enhancement and inhibition of activity^45,46^, and as fluctuations in resting-state networks^47^. This is in line with predictive processing frameworks in which predictions and prediction errors are exchanged across levels in cortical hierarchies, with a special role for regions such as the hippocampus in contextualizing this exchange^5,16^. While ripples represent possible outcomes, there is mixed evidence regarding whether they influence subsequent behavior^48–50^. Notably, the functional role of ripples is often examined by using navigational tasks in rodents, which heavily tap episodic memory and are biased by the provision of reward at the goal location. In humans, although there is emerging evidence for ripples supporting memory recall processes^51–53^, it remains unclear to what extent they have a role in prediction, in the absence of memory demands.

**Fig. 1:**
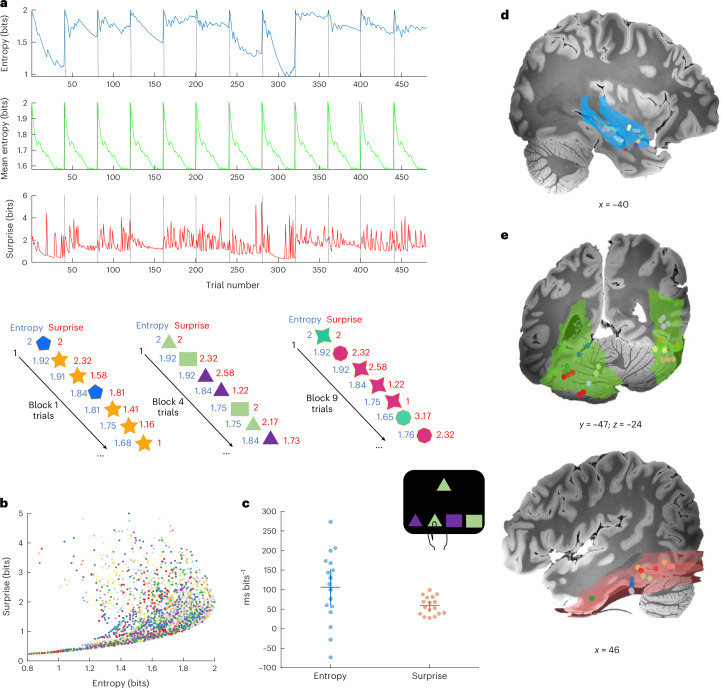
Task design and electrode contact localization. **a**, Distribution of entropy, mean entropy and surprise values across trials for one example participant. Bottom, example trials from 3 of 12 different blocks, and their associated entropy (blue) and surprise (red) values. Please note that the four colored shapes presented were unique to each block, with the probability of occurrence of these four stimuli varying between blocks. Participants were asked to perform a visuomotor selection task, choosing the corresponding colored shape from four alternatives with a button press, as depicted for block 4 in the current example. **b**, Correlation between entropy and surprise values across all patients. Each patient is represented by a color. By design, entropy and surprise are decorrelated, with high surprise occurring in the context of low entropy. For a comparison of model fits showing a linear correlation (*r* = 0.3) is inferior to nonlinear models, see Supplementary Note—Relationship between entropy and surprise. **c**, Group average (*n* = 17 patients) RT as an increase in ms per bit of information-theoretic measure. Error bars reflect 95% CI. **d**,**e**, Illustration of contacts in the hippocampus (smoothed hippocampus mask from the AAL atlas for visualization; blue), fusiform (red) and occipital cortex (green) overlaid on a 100-μm T1 scan of an ex vivo human brain, acquired on a 7T MRI scanner (https://openneuro.org/datasets/ds002179/versions/1.1.0); each color represents a patient. See Supplementary Fig. 1 for individual patients’ contacts. Unless otherwise stated, error bars represent the s.e.m. AAL, Automated Anatomical Labeling.

We hypothesized that high-order predictions—namely, predictions of predictability or precision—would be generated by the hippocampus before stimulus presentation, as a function of uncertainty (that is, predictability), and subsequently modulate cortical processing, or lower-level prediction errors. Ripples are a promising candidate to mediate the requisite precision weighting of prediction errors. We tested this hypothesis using intracranial local field potential recordings from patients with epilepsy to examine how the hippocampus and ventral visual stream regions (the occipital cortex and the fusiform gyrus) implement predictive processing. We used a simple paradigm—free of any explicit demand on episodic memory—in which participants were presented with a sequence of colored shapes and performed a visuomotor selection task, given a target stimulus and four options presented on-screen simultaneously. The probability distribution of stimulus presentation varied across task blocks, allowing for the quantification of stimulus-bound information-theoretic measures of entropy (that is, uncertainty or unpredictability of an outcome before it occurs) and self-information (that is, surprise or violation of predictions reflecting the improbability of a particular event) within each block. One might ask, how one can disambiguate the effects of surprise and entropy if—in the absence of entropy—there can be no surprise? The answer is straightforward—surprise is an attribute of a particular event, whereas entropy is an attribute of events that could happen. In other words, the surprise or self-information of an outcome scores the degree to which the event was ‘predicted’, whereas entropy scores the ‘predictability’ of an event before it is observed. Technically, entropy is the time average or an integral of surprise. This means entropy is the change in surprise and, therefore, over time, surprise and entropy are decorrelated (Fig. 1b). In the context of the current task, they can therefore be dissociated with respect to stimulus onset.

## Results

Seventeen participants successfully completed the task, with trial-by-trial measures of entropy and surprise modulating reaction time (RT) in accordance with Hick’s law^54^. Replicating results from this task in healthy adults^20^, participants’ RTs increased significantly per bit of surprise (*t*(16) = 10.61, *P* < 0.001, Cohen’s *d* = 2.57) and entropy (*t*(16) = 4.86, *P* < 0.001, Cohen’s *d* = 1.18; Fig. 1c).

### Prestimulus ripples increase with entropy

**Fig. 2:**
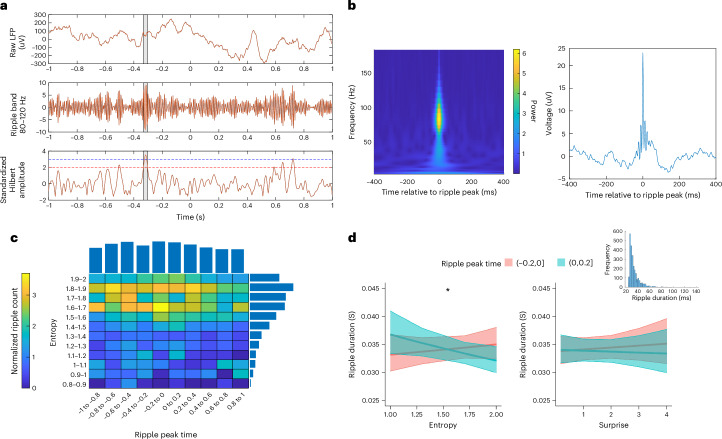
The frequency and duration of hippocampal ripples increase with uncertainty or expected information gain. **a**, An example ripple detected using the method discussed in ref. ^52^, showing the raw LFP trace (top), the ripple band signal (middle) and the standardized envelope (bottom). **b**, Grand average peri-ripple wavelet spectrogram (left) and raw field potential centered on ripple peak (*n* = 2,728 ripple events from 17 participants). **c**, Normalized ripple distribution across peri-stimulus time and entropy levels, accounting for the total number of trials in each entropy bin. Normalized ripple count for each time and entropy bin is color coded; average counts for each time and entropy bin are plotted as bars above and to the right of the 2D distribution, respectively. The majority of ripples occurred prestimulus and in high (but not highest) entropy levels (see Extended Data Fig. 3 for peri-stimulus ripple distribution). **d**, Ripple duration as a function of peri-stimulus time and information-theoretic measures. Ripple duration increased with entropy (that is, expected information gain for prestimulus ripples), but decreased for poststimulus ripples; result from mixed-effects gamma regression and subsequent omnibus *χ*^2^ Wald test for the interaction *P* = 0.0416). Ripple duration histogram, overall recorded ripples, is shown above right. Lines represent the mean and shaded areas represent the 95% CI. *P < 0.05 (two tailed). See Extended Data Fig. 1 for replication using a different ripple detection method^51^. LFP, local field potential.

**Extended Data Fig. 1:**
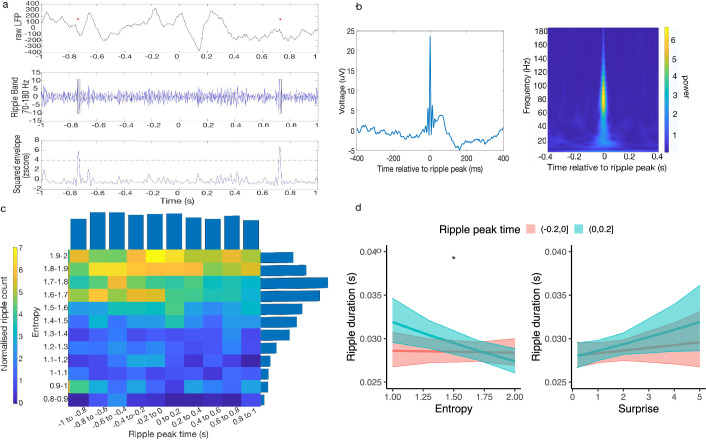
Hippocampal ripples detected using an alternative algorithm. **a**, An example ripple detected using the method discussed in ref. ^51^ showing the raw LFP trace (top), the ripple band signal (middle) and the standardized envelope (bottom). **b**, Grand average raw field potential centered on ripple peak (left) and peri-ripple wavelet spectrogram (n = 4653 ripple events from 17 participants). **c**, Normalized ripple distribution across peri-stimulus time and entropy levels, accounting for the total number of trials in each entropy bin. Normalized ripple count for each time and entropy bin is color-coded; average counts for each time and entropy bin are plotted as bars above and to the right of the 2D distribution, respectively. The majority of ripples occurred prestimulus and in high entropy levels. **d**, Ripple duration as a function of peri-stimulus time and information-theoretic measures. Ripple duration increased with entropy (that is, expected information gain) for prestimulus ripples, but decreased for poststimulus ripples (χ^2^(1) = 4.54, p = 0.032). Lines represent the mean, and shaded areas represent the 95% confidence intervals. *p < 0.05.

In view of a possible generative role for hippocampal ripples in predicting future events, we tested whether ripple occurrence was associated with increased uncertainty. Using a previously established ripple detection method^52^, a total of 2,728 hippocampal ripples were detected in all participants while they performed the visuomotor task (Fig. 2a and Extended Data Fig. 1). On examining the two-dimensional (2D) distribution of ripple peak time as a function of peri-stimulus time and entropy (Fig. 2b), we found a large proportion of ripples occurred in high entropy (1.7–1.9 bits) trials between −800 and −400 ms prestimulus. There was also a peak in ripple probability just before the stimulus onset (−200 to 0 ms) for entropy values from 1.6–1.7 to 1.8–1.9 bits.

**Extended Data Fig. 2:**
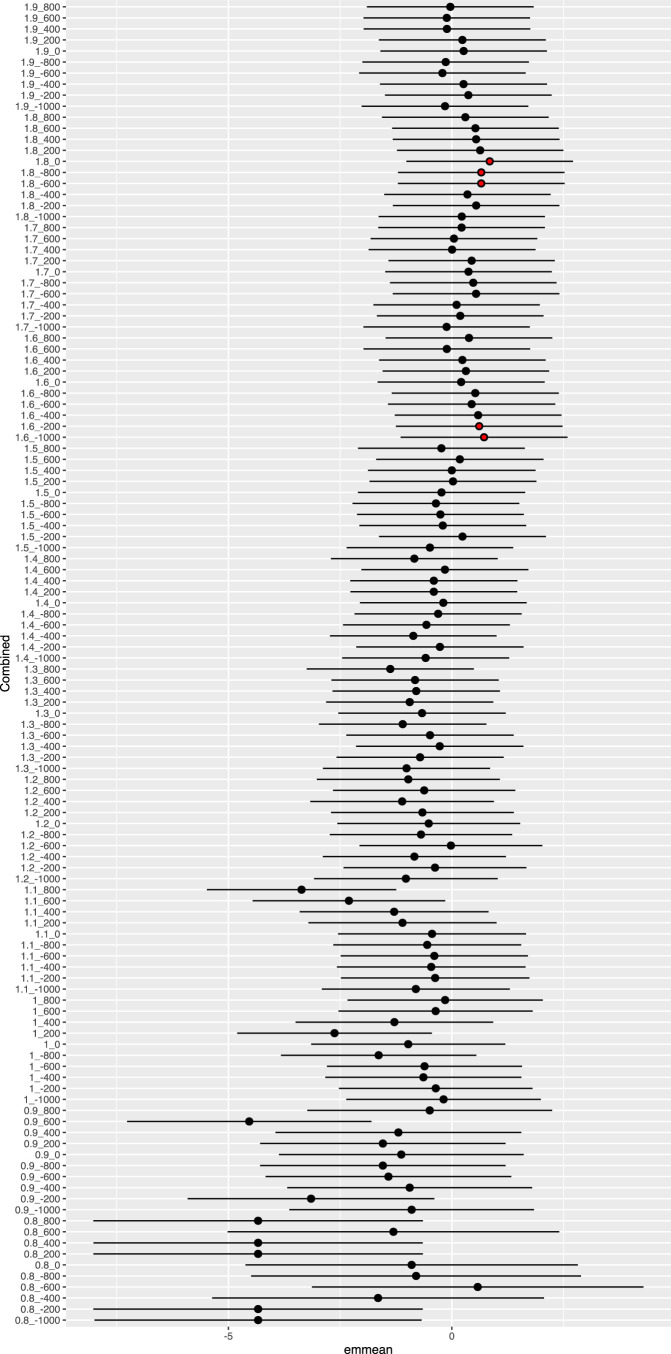
Estimated marginal means of the 2D ripple count as a function of entropy and time bins. The top 5 estimated marginal means (emmean) are marked in red, error bars represent upper and lower 95% confidence interval of the estimated marginal mean of the model (N = 17 patients).

**Extended Data Fig. 3:**
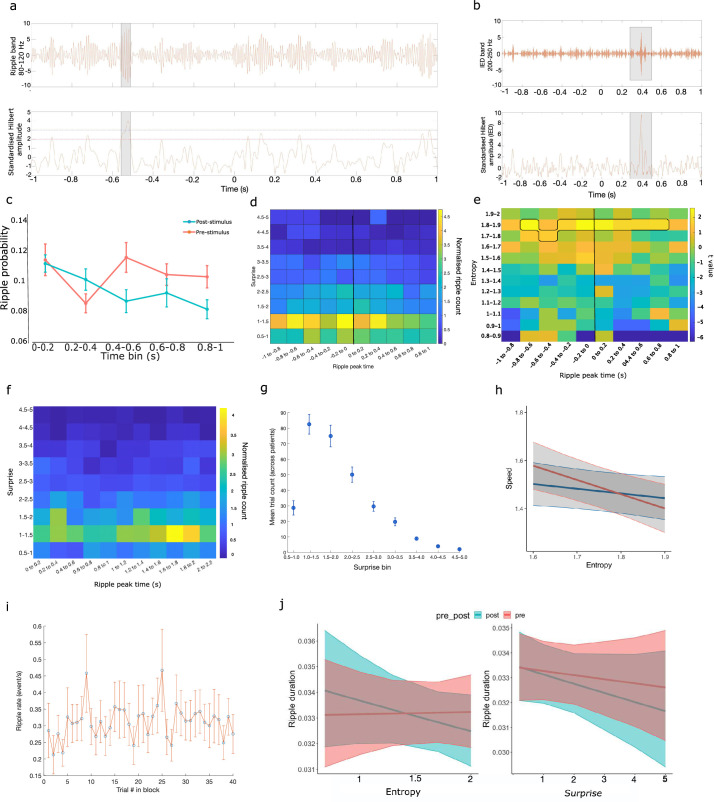
Hippocampal ripples. **a**, An example of ripple detection, showing the filtered ripple band and Hilbert amplitude thresholds. Gray shading indicates ripple duration. **b**, An example interictal epileptiform discharge (IED). **c**, Ripple frequency as a function of time bin and peri-stimulus time; a main effect of peri-stimulus time (F(1,16) = 6.02, p = 0.026), with more ripples observed prestimulus. Error bars represent standard error of the mean (N = 17 patients). **d**, Normalized distribution of ripples as a function of peri-stimulus time and level of stimulus-bound surprise. **e**, Cluster-based statistical test on the normalized ripple count in the 2D entropy-time histogram using TFCE revealed an extended cluster (−800 ms to +800 ms) of increased ripple count for high levels of entropy, with peaks around −800 to −600 ms prestimulus, −200 to 0 and 0 to 200 ms poststimulus. Color bar indicates t-value. **f**, Poststimulus ripple rate as a function of surprise (normalized by the number of trials within each surprise bin). If entropy-associated prestimulus ripples were instead reflecting surprise-associated postprocessing of the previous stimulus, an increase in ripple count should be observed for high surprise later in the trial. When locked to stimulus onset and going forward in time (up to 2.2 s poststimulus onset, which corresponds to the time between presentation of successive stimuli), such an increase was not observed, that is, the ripple rate in the top right quadrant of the plot shows a low ripple rate. Instead, there is an increase in ripple count for low surprise (1–1.5 bits) at later time bins. This designation of 1–1.5 bits as ‘low surprise’ is mathematically determined by our task design (maximum surprise ~5 bits, minimum approaching 0). This range of surprise values corresponds, on average, to a value of 1.67 bits of entropy. This plot is, therefore, consistent with the increase in ripple count in the prestimulus period for upcoming entropy values around 1.6 to 1.9 bits. Thus, when we plot ripple occurrence as a function of surprise, we observe no increase in ripple count for high surprise trials in the poststimulus period. Instead, ripples cluster precisely following low-surprise trials, very late in the trial (which would correspond to prestimulus bins of the next trial). **g**, Distribution of surprise values in the task (error bars depict s.e.m.; N = 17 patients). **h**, Speed (1/RT) as a function of prestimulus ripple status and entropy. For trials with a prestimulus ripple there was a faster response, more pronounced (at a trend level) for entropy levels around 1.6 to 1.8 bits. Error bars represent the standard error of the mean. **i**, Ripple rate as a function of trial in block (N = 17 patients), error bars represented standard error of the mean. **j**, Ripple duration as a function peri-stimulus time and information-theoretic measures. Entropy by ripple peak time interaction (χ^2^(1) = 0.66 p = 0.41) and surprise by ripple peak time interaction (χ^2^(1) = 0.17, p = 0.678). Lines represent the mean and shaded area the 95% CI.

We tested for the significance of this observation in several ways. First, a two-sample Kolmogorov–Smirnov test showed a significant difference between the ripple distribution and a uniform distribution with the same minimum and maximum counts (*k* = 0.241, *P* = 0.001). Next, we performed a Spearman’s correlation between the observed and permuted distributions to ensure that ripple distribution was not correlated with random noise. The correlation between the two was computed in every permutation (1,000 permutations in total) per participant, and the mean correlation across participants was compared to 0 using a one-sample *t* test. The observed 2D distribution was not correlated with the random permutations (*t*(16) = 1.24, *P* = 0.23). Third, we used a mixed-effects gamma generalized linear model (GLM) on the normalized count values to identify the bins showing the largest number of ripples, using entropy and time bin as predictors. We found a significant interaction between entropy and ripple peak time (*χ*^2^(99) = 207.6, *P* < 0.001), with the five largest estimated marginal means identified around −1,000 to –400 ms and 1.6 to 1.8 entropy bins, as well as just before stimulus onset (−0.2 to 0, entropy values of 1.8–1.9) and just after stimulus onset (0–0.2, entropy values of 1.8–1.9). This is in line with the visual representation of the 2D distribution shown in Fig. 2c (all estimated marginal mean values are shown in Extended Data Fig. 2). Next, we examined ripple frequency as a function of time bin and peri-stimulus time using a two (pre/post) by five (time bin) analysis of variance. We found a main effect of peri-stimulus time (*F*(1.16) = 6.02, *P* = 0.026), with more ripples observed prestimulus (Extended Data Fig. 3c), whereas the main effect of bins and the interaction were not significant (all *P*s > 0.073). Finally, a threshold-free cluster enhancement (TFCE) analysis of the 2D histogram showed significant effects again primarily in the entropy range of 1.8–1.9 bits, albeit for a longer time period (Extended Data Fig. 3e).

In view of a proposal^55^ that awake replay during SWRs increases following prediction error (that is, surprise), we then provide evidence that human hippocampal ripple frequency is increased by unpredictability and not surprise. If ripples reflected processing of prediction errors postpresentation of surprising stimuli, rather than the predictability of upcoming stimuli, one would expect to see an increased ripple rate with surprise. However, the 2D distribution of ripple peak time as a function of peri-stimulus time and surprise (Extended Data Fig. 3d) shows cluster of ripples at low surprise (at 1–1.5 bits). Next, to further demonstrate that the increased ripple count observed in the prestimulus time bin is not driven by a surprise effect, we applied a Poisson GLM predicting ripple counts as a function of entropy, surprise and mean entropy for each time bin. The *β* values across participants were then tested against zero in a two-tailed *t* test using a cluster-based Monte Carlo permutation to correct for multiple comparisons as implemented in FieldTrip. In line with the analyses reported above, we found a significant effect in the −800 to −600 ms time bin such that ripple count increased with entropy (cluster *t* = 2.66, *P* = 0.009, 95% confidence interval (CI) = 0.002). No other significant effects were observed for entropy. For surprise, we found a negative association with ripple count in the same time bin (−800 to −600 ms; *t*(16) = −1.89, *P* = 0.039, uncorrected), indicating that as surprise increases, there is a decrease in ripple count for this time window (note the effect of surprise did not survive a correction for multiple comparisons). This analysis confirms that the increased prestimulus ripple count is related to entropy, as opposed to the effect of surprise, and demonstrates statistical independence—we observed significant effects of entropy on ripple counts, but no significant effects of surprise across all time bins (Extended Data Fig. 2). Finally, we analyzed data from an independent control experiment^56^ using a classical ‘oddball’ paradigm (Supplementary Note (Independent data from oddball task) and Supplementary Table 2). Participants were presented with lists of words where occasional perceptually distinct stimuli (perceptual oddballs) appeared in a different font among standard control words. These oddball stimuli were highly surprising by design (that is, this is a standard paradigm for eliciting prediction errors), yet we observed no increased ripple count for oddball stimuli compared to control words (Supplementary Fig. 2 and Supplementary Table 3). The dissociation between oddball surprise (no ripple increase) and entropy-based uncertainty (clear ripple increase) supports our interpretation that ripples encode predictions about predictability rather than responses to unpredicted events.

The duration of ripples has been shown to be functionally relevant for memory consolidation in rodents^57^. In predictive coding formulations, this rests on the augmentation of associative (that is, activity or experience dependent) plasticity by precision weighting that increases presynaptic (prediction error) afferents^4,58^. To test for changes in ripple duration with uncertainty, we then examined the relationship between ripple duration and the information-theoretic measures. As ripple duration followed a right-skewed distribution (Fig. 2d), a mixed-effects gamma regression was used. First, we tested whether ripple duration was modulated by peri-stimulus time, entropy and surprise. We did not observe any significant main effects (all *P*s > 0.381) or interactions (time window by entropy—*χ*^2^(1) = 0.665, *P* = 0.414; time window by surprise—*χ*^2^(1) = 0.171, *P* = 0.679; Extended Data Fig. 3j). However, comparing ripples occurring just before (−200 to 0 ms, where there was an increase in ripple frequency; Fig. 2b) and the corresponding period just after the stimulus (0 to 200 ms) revealed a significant main effect of peri-stimulus time window (*χ*^2^(1) = 5.04, *P* = 0.0248), as well as an interaction between time window and entropy (*χ*^2^(1) = 4.15, *P* = 0.0416; Fig. 2d). This interaction suggests that as entropy increased, prestimulus ripple duration increased, but decreased for poststimulus ripples. Ripple duration was again not modulated by surprise (main effect—*χ*^2^(1) = 0.279, *P* = 0.597; interaction—*χ*^2^(1) = 0.31, *P* = 0.577), suggesting that ripple duration reports predictability (that is, expected information gain) as opposed to violations of predictions (that is, observed information gain).

### RT as a function of ripples

Given that there is a relationship between entropy and RT^20,54^, as well as between ripples and entropy, we examined whether the presence of ripples modulates the relationship between RT and entropy—and surprise—on a trial-by-trial basis. To ensure that sufficient prestimulus ripple trials were included, this analysis was restricted to trials with 1.6–1.9 bits of entropy, where most prestimulus ripples were observed (50% prestimulus ripple trials and 50% no ripple trials are within these values). We found a significant main effect of ripple status, with faster responses in trials with a prestimulus ripple (*χ*^2^(1) = 4.33, *P* = 0.037). This is an important observation because the precision of prediction errors corresponds to the rate of evidence accumulation that underwrites reaction speed. In other words, prediction errors that are afforded more precision exert their effects on belief updating more rapidly. There was also a main effect of surprise, with faster responses for lower levels of surprise (*χ*^2^(1) = 91.3, *P* < 0.001), as per Hick’s law. The interaction between ripple status and surprise, however, was not significant (*χ*^2^(1) = 2.58, *P* = 0.107), indicating that ripples did not modulate the relationship between speed and surprise. There was a trend toward an interaction between entropy and ripple status (*χ*^2^(1) = 3.41, *P* = 0.064), with simple slopes of entropy in trials without ripples (estimate = 0.2, *t*(16) = 1.55, *P* = 0.12) and in trials with prestimulus ripples (estimate = 0.59, *t*(16) = 3.28, *P* = 0.001), indicating a steeper positive slope in trials with prestimulus ripples.

### Region-specific responses to uncertainty

**Fig. 3:**
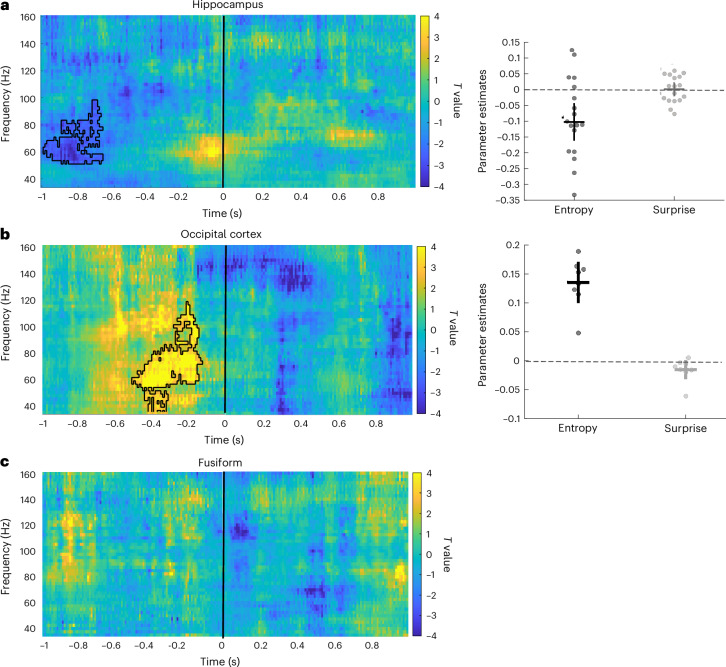
Hippocampal and occipital cortical gamma activity is modulated by entropy ‘before’ stimuli are presented. **a**, Left, time-resolved spectral power in the hippocampus (*n* = 17 patients) shows a significant negative association between gamma power and entropy around 800 ms prestimulus (significant permutation-based cluster from one-sample two-tailed *t* test against a value of 0 outlined in black here and in Figs. 4 and 5). During highest entropy levels, gamma power was lowest (see Extended Data Fig. 5 for gamma power as a function of trial in block). No significant clusters were found in the poststimulus time window. Right, parameter estimates for each predictor within the significant cluster; each point pertains to one patient, horizontal bars represent the group mean and vertical error bars represent 95% CI. **b**, In occipital cortex (*n* = 8), there was an increase in gamma power as entropy increased, around 400 ms prestimulus. Again, entropy was not associated with changes in the poststimulus time windows. Each point pertains to one patient, horizontal bars represent the group mean and vertical error bars represent 95% CI. **c**, In the fusiform cortex (*n* = 7), no effects of entropy were observed either prestimulus or poststimulus.

**Extended Data Fig. 4:**
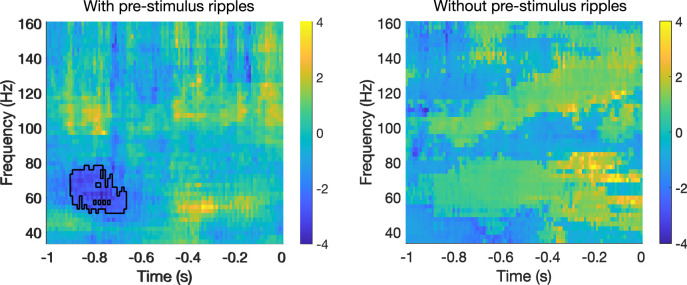
Prestimulus hippocampal response to entropy split by whether a prestimulus ripple was present. Examining the hippocampal response to entropy on trials in which a prestimulus ripple was present, compared to trials without a prestimulus ripple, we found a negative association between hippocampal gamma and entropy in trials with a prestimulus ripple (around the same time and frequencies as reported in Fig. 3a). Cluster t = −488.1, p = 0.0248 95% CI = 0.0043.

**Extended Data Fig. 5:**
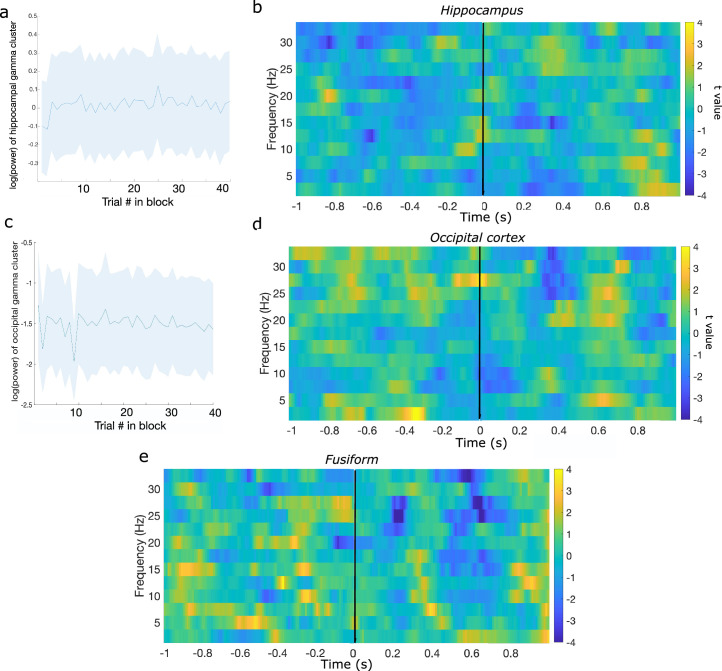
Entropy-related responses. **a**, Log-transformed gamma power in hippocampal prestimulus entropy cluster (shown in Fig. 3a), as a function of trial in block. Shaded areas represent standard error of the mean. **b**, Hippocampal lower frequencies entropy responses, prestimulus and poststimulus. **c**, Log-transformed gamma power in occipital cortex prestimulus entropy cluster (shown in Fig. 3b), as a function of trial in block. Shaded areas represent standard error of the mean. **d**,**e**, Occipital (**d**) and fusiform (**e**) cortex lower frequencies entropy responses, prestimulus and poststimulus.

To further characterize hippocampal and cortical correlates of entropy and surprise, we then examined time-resolved spectral responses as a function of these measures. The focus was on two cortical regions—the fusiform gyrus, previously shown with functional magnetic resonance imaging (fMRI) to respond to surprise in this task^20^, and occipital cortex, lower down in the visual cortical hierarchy than the fusiform and putatively supplying it with bottom-up information (for example, prediction errors). Hippocampal activity in the gamma range (57.5–97.5 Hz; Fig. 3a) was negatively associated with entropy in the prestimulus period, around −990 to −670 ms prestimulus (summed cluster *t* = −762.8, *P* = 0.0218; smallest *t* value in cluster = −4.57, *P* = 0.0218, Cohen’s d = 1.08), showing reduced gamma power preceding more uncertain outcomes (see Extended Data Fig. 4 for split by the presence of prestimulus ripple). This time window partly overlaps with peaks in ripple occurrence, although, notably, the gamma power around this time window was reduced under high entropy. Further examination of the negative association with entropy, as a function of trial in block, showed that the reduction in gamma power was mostly concentrated in the first few trials of the block (Extended Data Fig. 5), before the emergence of prestimulus ripples.

In the occipital cortex, on the other hand, a positive association between gamma activity (35–117.5 Hz; Fig. 3c) and entropy was observed around −510 to −130 ms prestimulus (summed *t* = 1,694.1, *P* = 0.0078; largest *t* value in cluster = 10.67, *P* = 0.0078, Cohen’s *d* = 3.77). No significant responses to entropy were found in the fusiform, or in the poststimulus time period for all three areas (Fig. 3). Under the simplifying assumption that gamma activity reflects the amplitude of precision-weighted prediction errors, these results are consistent with an increase in the precision of visual prediction errors, with a concomitant decrease in the precision of hippocampal prediction errors, before stimuli with higher expected information gain. This could be read as instantiating the right kind of attentional set, when salient information can be anticipated in advance^59^.

### Ripple-triggered cortical responses

**Fig. 4:**
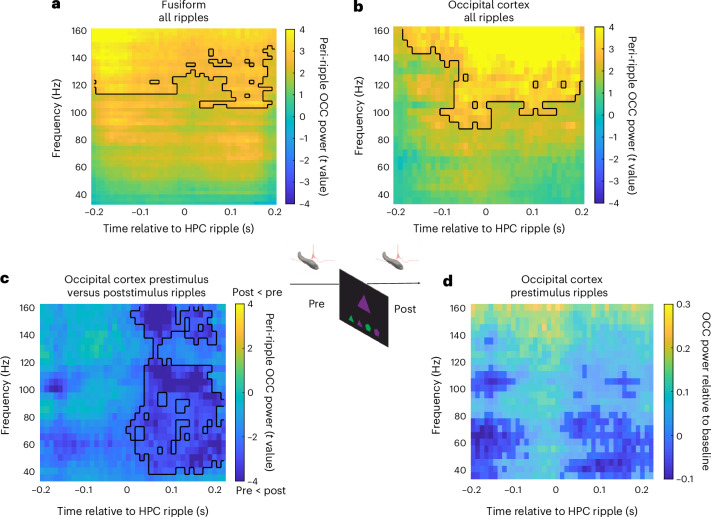
Peri-hippocampal ripple cortical activity. **a**,**b**, Cortical TF analyses time locked to the hippocampal ripple peak time. Across all ripple trials, there was an overall increase in high gamma power in the fusiform (**a**) and occipital cortex (**b**) time locked to hippocampal ripple. **c**, Comparing trials with prestimulus versus poststimulus hippocampal ripples, there was a reduction in occipital gamma power in trials with prestimulus ripples, compared to poststimulus ripples. **d**, Further examination of this effect revealed that it was driven by a suppression of gamma power relative to baseline in trials with prestimulus hippocampal ripples and compared to trials with no ripples (Extended Data Fig. 6). See Extended Data Fig. 7 for replication using a different ripple detection method. OCC, occipital cortex; HPC, hippocampus.

**Extended Data Fig. 6:**
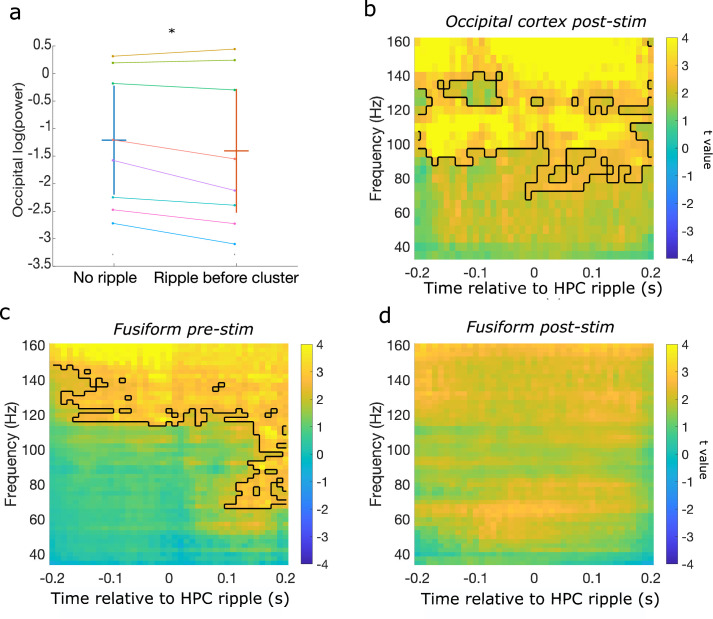
Peri-ripple cortical modulation. **a**, Mean of log power in occipital cortex cluster (N = 8) positively associated with entropy in the prestimulus time window, split by whether there was a hippocampal ripple just before the significant effect or if there was not, two-tailed paired t-test (t(7) = 2,51, p = 0.0401, CI = (0.011, 0.377)); each point pertains to one patient, horizontal bars the group mean, and vertical error bars represent 95% confidence interval. Occipital gamma power was suppressed when there was a hippocampal ripple compared to when there was not. **b**, T-statistics of peri-ripple activity in occipital cortex for poststimulus ripples. **c**,**d**, Peri-ripple activity in the fusiform for (**c**) prestimulus and (**d**) poststimulus ripples. *p < 0.05.

**Extended Data Fig. 7:**
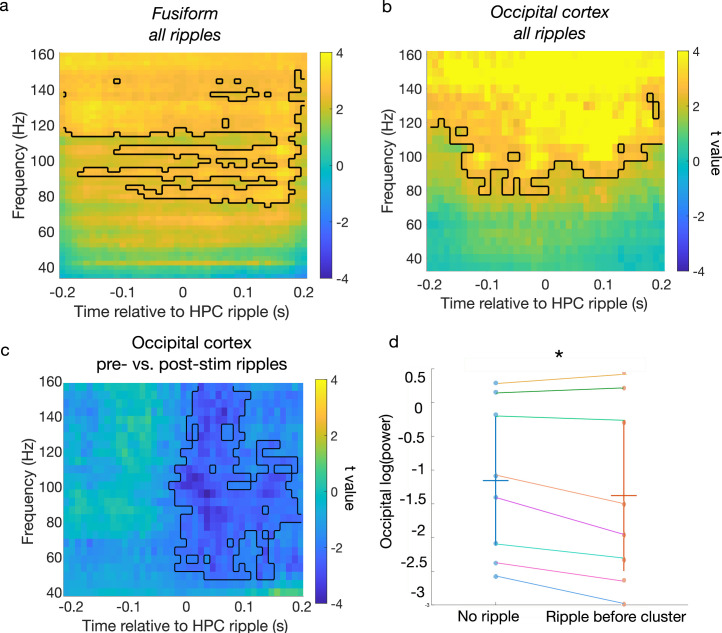
Peri-ripple cortical modulation using an alternative detection algorithm. **a**,**b**, Cortical time-frequency analyses time-locked to the hippocampal ripple peak time, detected using the algorithm discussed in ref. ^51^. Across all ripple trials there was an overall increase in high gamma power in the fusiform (**a**) and occipital cortex (**b**) time-locked to hippocampal ripple. **c**, Comparing trials with prestimulus versus poststimulus hippocampal ripples, there was a reduction in occipital gamma power in trials with prestimulus ripples, compared to poststimulus ripples. **d**, Mean of log power in occipital cortex cluster positively associated with entropy in the prestimulus time window, split according to whether there was a hippocampal ripple just before the significant effect or if there was not (N = 8), each point pertains to one patient, horizontal bars the group mean, and vertical error bars represent 95% confidence interval. Occipital gamma power was suppressed when there was a hippocampal ripple compared to when there was not; dependent t-test t(7) = 2.54, p = 0.038, Cohen’s d = 0.9. *p < 0.05 (two-tailed).

Next, we investigated whether ripple occurrence instates a temporal relationship between the hippocampus and visual processing regions in which prestimulus modulations of gamma activity were also observed as a function of entropy. First, we compared peri-ripple cortical time–frequency (TF) responses, dichotomized by whether the hippocampal ripple occurred before or after stimulus onset (Fig. 4a,b). We found an overall increase in high gamma power in the fusiform (−200 to 200 ms peri-ripple, 105–160 Hz, summed cluster *t* = 2,171.1, *P* = 0.0156; largest *t* value in cluster = 4.44, *P* = 0.0156, Cohen’s *d* = 1.67) as well as in occipital cortex (−180 to 200 ms peri-ripple, 95–160 Hz, summed cluster *t* = 1,474, *P* = 0.0039; largest *t* value in cluster = 9.15, *P* = 0.0039, Cohen’s *d* = 3.27) locked to hippocampal ripples. Furthermore, in occipital cortex, prestimulus and poststimulus ripples modulated activity differently (−10 to 200 ms peri-ripple, 35–160 Hz; Fig. 4c; summed *t* = −1,094.5, *P* = 0.0039; smallest *t* value in cluster = −8.99, *P* = 0.0039, Cohen’s *d* = 3.17). Specifically, occipital gamma activity was reduced after prestimulus ripples compared to poststimulus ripples. This effect was partly driven by a power suppression, compared to a baseline period, of occipital gamma around prestimulus ripples (Fig. 4d shows raw power suppression in relation to baseline). That is, when examining occipital gamma activity in the prestimulus period in which a positive association with entropy was found (indicating an overall power increase), there was reduced gamma power in trials with a hippocampal ripple in the period before the significant cluster compared to trials with no ripples (*t*(7) = 2.51, *P* = 0.040, Cohen’s *d* = 0.889; Extended Data Fig. 6a). Taken together, these findings are indicative of a hippocampal prestimulus ripple-induced suppression of prestimulus gamma power in the occipital cortex (see Extended Data Fig. 7 for replication using a different ripple detection method). No differences were observed between prestimulus and poststimulus ripple modulation of fusiform activity, or in lower frequencies in either cortical region.

Next, we examined whether ripple-modulated cortical activity was correlated with entropy or surprise. There were no significant associations between peri-ripple cortical activity and either information-theoretic measure, suggesting that the hippocampal ripple modulation of cortical response was not affected by uncertainty or surprise, and thus ripples may act as a general mechanism for hippocampal modulation of cortical activity, with context dependence of this modulation affected by ripple rate and/or duration.

### Hippocampal and fusiform gamma to surprise

**Fig. 5:**
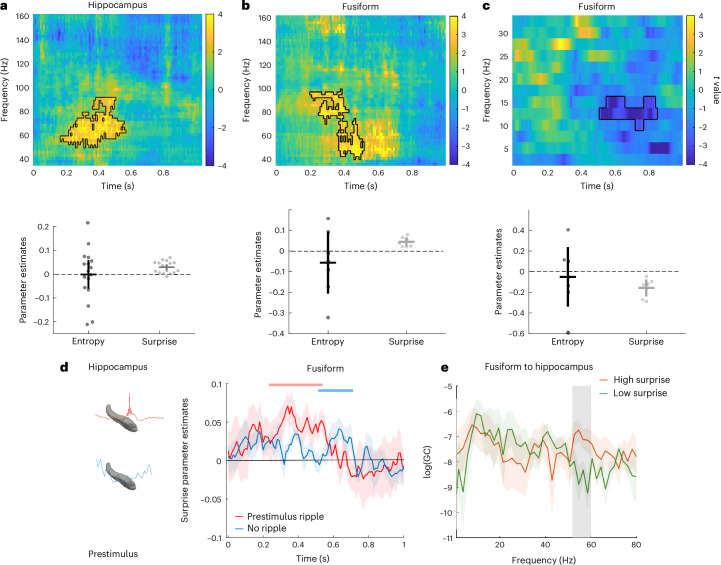
Surprise-driven responses. **a**, Hippocampal increase in gamma power as surprise increases. Bottom, parameter estimates for each predictor within the significant cluster (*n* = 17). Each point pertains to one patient, horizontal bars represent the group mean and vertical error bars represent 95% CI. **b**, Fusiform gamma power increases as surprise increases (*n* = 7). Each point pertains to one patient, horizontal bars represent the group mean and vertical error bars represent 95% CI. **c**, Fusiform beta oscillations decrease as surprise increases (*n* = 7). Each point pertains to one patient, horizontal bars represent the group mean and vertical error bars represent 95% CI. See Extended Data Fig. 9 for low-frequency responses to surprise. **d**, The increase in fusiform gamma power of 40–95 Hz (extracted from significant cluster) occurs earlier in trials with prestimulus hippocampal ripples (red) compared to trials without ripples (blue). Lines represent the mean, and shaded areas represent the s.e.m. See Extended Data Fig. 8 for replication using a different ripple detection method. **e**, Directed information flow from fusiform to hippocampus for high (orange) versus low (green) surprise trials was observed in the gamma band (shaded rectangle). Lines represent the mean, and shaded areas represent the s.e.m.

**Extended Data Fig. 8:**
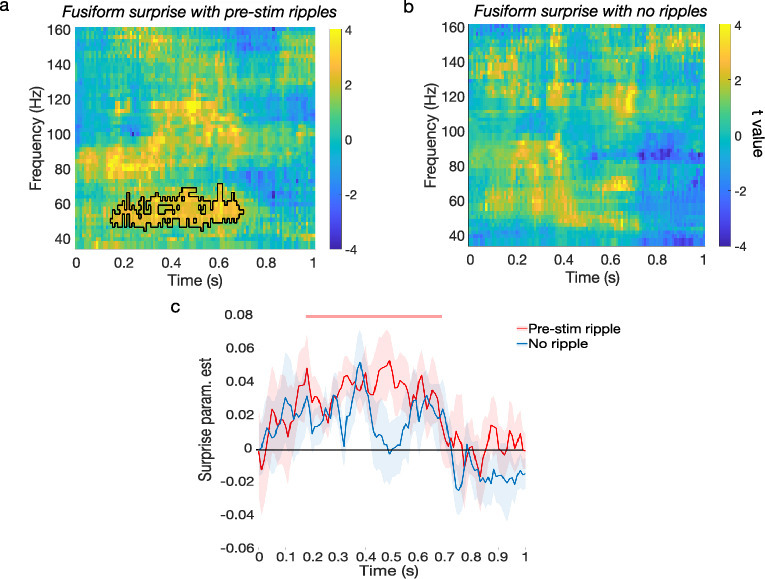
Hippocampal prestimulus ripples modulate fusiform response to surprise using ripples detected with an alternative algorithm. **a**,**b**, Fusiform poststimulus association with surprise in trials with (**a**) prestimulus ripple compared to (**b**) without prestimulus ripples (no significant response to surprise observed). **c**, The increase in fusiform gamma power 40–95 Hz (extracted from significant cluster in trials with prestimulus hippocampal ripples (red) compared to trials without ripples (blue). Ripples detected using the algorithm described in ref. ^51^. Lines represent the mean, and shaded areas the standard error of the mean.

**Extended Data Fig. 9:**
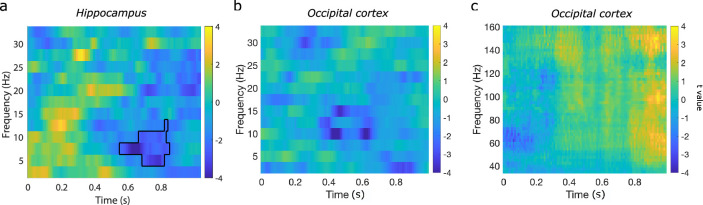
Surprise responses. **a**, Hippocampal low frequency response to surprise. **b**,**c**, Occipital cortex (**b**) low and (**c**) high frequency responses to surprise.

We expected that prediction error, in the form of surprise, would elicit hippocampal and cortical responses. In line with previous findings, a positive association between surprise and hippocampal activity in the gamma range (52.5–90 Hz; Fig. 5a and see Extended Data Fig. 8 for replication using a different ripple detection method) was observed around 160–540 ms poststimulus (summed cluster *t* = 888.2, *P* = 0.016; largest *t* value in cluster = 4.91, *P* = 0.016, Cohen’s *d* = 1.2) and a negative association between surprise and hippocampal activity in theta band (5–12.5 Hz; Extended Data Fig. 9a) later in the trial (530–810 ms poststimulus; summed cluster *t* = −169.4, *P* = 0.011; smallest *t* value in cluster = −3.19, *P* = 0.011, Cohen’s *d* = 0.773) in line with previous observations^23^. In the fusiform, there was a positive association between surprise and gamma activity (40–95 Hz; Fig. 5b) around 200–510 poststimulus (summed cluster *t* = 741.9, *P* = 0.0234; largest *t* value in cluster = 6.79, *P* = 0.0234, Cohen’s *d* = 2.56), and a negative association between surprise and alpha/beta power (10–15 Hz; Fig. 5c) around 510–840 ms poststimulus (summed cluster *t* = −199.5, *P* < 0.001; largest negative *t* value in cluster = −5.41, *P* < 0.001, Cohen’s *d* = 2.05). No significant associations between surprise and occipital activity were observed.

### Ripple-modulated responses to surprise

**Extended Data Fig. 10:**
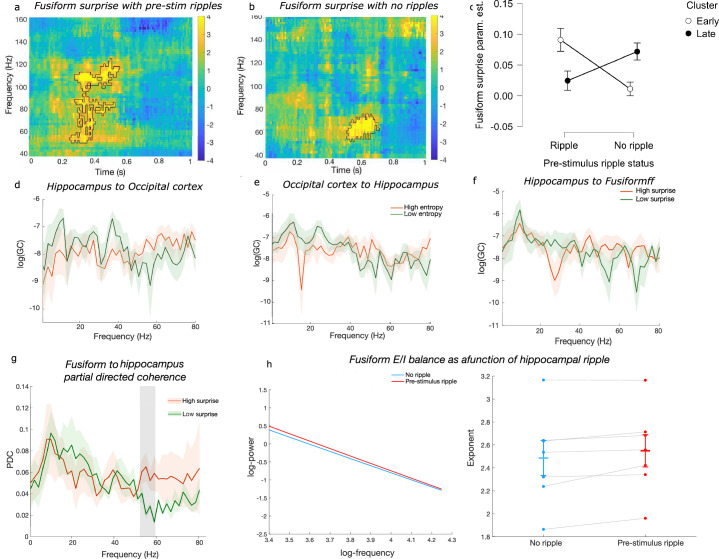
Fusiform poststimulus response to surprise is modulated by prestimulus hippocampal ripples and directed cortical-hippocampal information flow. **a**,**b**, Fusiform poststimulus association (**a**) with surprise in trials with prestimulus ripple earlier than (**b**) without prestimulus ripples. **c**, Double-dissociation of fusiform gamma response to surprise (early and late clusters) in the presence and absence of a prestimulus hippocampal ripple (n = 7). Error bars represent the standard error of the mean, p = 0.004. **d**,**e**, Granger causality (GC) analysis between the hippocampus and occipital cortex for entropy. **f**, Granger causality from the hippocampus to fusiform for surprise. Shaded areas represent the standard error of the mean. **g**, Partial directed coherence (PDC) values for high (orange) versus low (green) surprise trials were significantly higher in the gamma band (shaded rectangle, p < 0.001). This is analogous to the results presented in Fig. 5e, using a Granger causal analysis. PDC was computed using the FieldTrip toolbox in Matlab. The median-split data were downsampled to 250 Hz, and PDC was calculated for frequencies ranging between 0 and 80 Hz with a smoothing of 10 Hz. Shaded areas represent the standard error of the mean. param. est.: parameter estimate of fusiform response to surprise. **h**, Trials without prestimulus ripples show smaller exponents (that is, flatter spectral slopes), indicating a shift toward excitation (E>>I), whereas trials with prestimulus ripples show steeper slopes, indicating a more balanced E/I state. Error bars represent the standard error of the mean (N = 7 patients).

Under the predictive processing framework, a cardinal function of neural activity is to minimize precision-weighted prediction error^5^. A possible role for hippocampal ripples is to broadcast the predicted precision of forthcoming prediction errors. Specifically, we hypothesized that prestimulus ripples would amplify precision-weighted prediction errors, evoked by stimuli in an unpredictable (high entropy) context. To test this hypothesis, we examined whether the occurrence of prestimulus hippocampal ripples modulated poststimulus (0–1 s) cortical responses to surprise (for example, the increase in gamma power in the fusiform). This hypothesis was confirmed in the fusiform cortex. We found an early (250–550 ms poststimulus, summed *t* = 936.9, *P* = 0.0078) increase in fusiform gamma power (47.5–122.5 Hz) in response to higher surprise in trials that were preceded by a prestimulus hippocampal ripple. By contrast, a later (510–710 ms) increase in gamma power (50–72.5 Hz, summed *t* = 473.8, *P* = 0.0078) was observed to higher surprise when there was no prestimulus ripple (Fig. 5d and Extended Data Fig. 10a,b). The presence of a faster fusiform response to surprise in trials with prestimulus ripples could simply reflect a co-occurrence of the two. To rule out this possibility, we examined whether there was a double dissociation of the surprise parameter estimate using a repeated measures analysis of variance with ripple status (no ripple, prestimulus ripple) and time window (early, later) as factors. This showed a significant interaction (*F*(1,6) = 19.6, *P* = 0.004, *η*^2^_*P*_ = 0.766), confirming the double dissociation (Extended Data Fig. 10c) such that an early effect of surprise was observed for trials with prestimulus ripples, but not for trials without ripples; conversely, a later surprise effect was found in trials without ripples, but not in trials with prestimulus ripples. This result suggests that the presence of a prestimulus hippocampal ripple modulates the fusiform response to surprise, such that it is faster—and of greater amplitude—compared to trials without a prestimulus ripple. The presence of prestimulus hippocampal ripples did not modulate poststimulus occipital responses to surprise.

This observation raised the question of what the impact of prestimulus hippocampal ripples could be on cortical dynamics in the fusiform that would later modulate prediction error gamma response. We therefore examined aperiodic exponents in the fusiform cortex (30–70 Hz, from −750ms to +250 ms around stimulus onset) as a measure of cortical excitation/inhibition (E/I) balance^60,61^. This analysis revealed that the aperiodic exponent—and hence the underlying cortical excitability state—differs significantly based on the occurrence of a prestimulus hippocampal ripple (*t*(6) = 2.63, *P* = 0.039). Specifically, trials without prestimulus ripples show smaller exponents (mean = 2.485, s.d. = 0.405; that is, flatter spectral slopes), indicating a shift toward excitation (E>>I), whereas trials with prestimulus ripples show steeper slopes (mean = 2.549, s.d. = 0.371), indicating a more balanced E/I state (Extended Data Fig. 10h). Critically, the most neurobiologically grounded accounts of precision encoding appeal to fast-spiking inhibitory interneurons see ref. ^16^ for a particular focus on the hippocampus). In this framework, the more balanced E/I state following ripples reflects increased inhibitory control, which directly implements higher precision weighting of prediction errors. Conversely, the E>>I state in trials without ripples reflects reduced inhibitory control and correspondingly lower precision weighting.

### Directed information flow

Finally, we examined information flow between the hippocampus and fusiform and occipital cortex by testing for Granger causality (GC) as a function of surprise and entropy. To do so, a median split for each information-theoretic measure was applied and a nonparametric spectral GC analysis was performed. Guided by the TF effects above, we focused on hippocampus–occipital cortex for entropy, and hippocampus–fusiform cortex for surprise, each centered in time around the observed TF effect (−1000 to −500 ms prestimulus for entropy, and 150 to 650 ms poststimulus for surprise). For surprise, we found increased bottom-up information flow (fusiform → hippocampus) in the gamma range (52–60 Hz; Fig. 5e and Extended Data Fig. 10a–c) for high versus low surprise (summed *t* = 13.2, *P* = 0.0078). To ensure that this effect was valid, we ran two further analyses—first, a GC analysis on the time-reversed data, showing an effect in the same frequency ranges in the opposite direction, as expected, and, second, a partial directed coherence analysis that revealed a significant information flow effect from the fusiform to the hippocampus in high versus low surprise trials for the same frequency range of 52–59 Hz (cluster *t*(6) = 4, *P* < 0.001; Extended Data Fig. 10g).

**Fig. 6:**
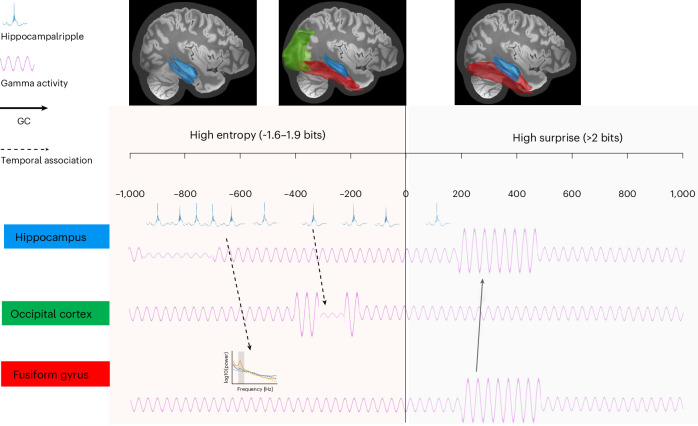
Human hippocampal–cortical dialog in precision weighting of prediction errors. Peri-stimulus time is indicated in ms, and involvement of relevant brain areas (hippocampus in blue, occipital cortex in green and fusiform cortex in red) at different time points is indicated by the inclusion of that structure in the top row. Before stimulus presentation (left), unpredictable visual stimulus streams modulate hippocampal gamma activity and ripple occurrence, with a change in dynamics in visual cortical areas temporally associated with hippocampal ripples. These effects are observed before stimulus presentation. From ~1,000 ms before stimulus presentation, there is a reduction in hippocampal gamma activity, coinciding with a peak in ripple rate at ~800 ms prestimulus. By contrast, occipital cortex shows an increase in gamma power ~500 ms prestimulus, but this increase is suppressed if the hippocampus generates a ripple. Spectral power in the fusiform cortex is not modulated by entropy prestimulus. However, the occurrence of a prestimulus hippocampal ripple (which is most frequent under high entropy) shifts the excitatory–inhibitory balance of the prestimulus fusiform field potential to a more inhibitory state. We interpret these prestimulus effects to reflect the setting of cortical dynamics to imbue precision to subsequently surprising stimuli in the high entropy context. In line with these predictions, different levels of entropy are not associated with any poststimulus changes in spectral power. Surprising stimuli, however, lead to poststimulus increases in gamma power in fusiform and hippocampus (right), with directed functional connectivity following fusiform to hippocampus in this gamma range. The critical observation is that a prestimulus hippocampal ripple is associated with a change in excitatory–inhibitory balance in the fusiform cortex (left), and a subsequent poststimulus gamma response in the fusiform to surprising stimuli (right) that is greater in amplitude and of shorter latency (~200 ms). No poststimulus responses to surprising stimuli are observed in occipital cortex, in keeping with an established role of higher-order visual cortices (that is, fusiform) in generating prediction error neuronal responses. For illustrative purposes, gamma activity is shown as a continuous oscillation.

Subsequent GC analyses showed no significant effects in the reverse direction (hippocampus → fusiform; Extended Data Fig. 10f). Thus, top-down effects are expressed as hippocampal ripple-dependent modulation of fusiform cortical dynamics ‘before’ surprise, whereas bottom-up effects manifest as fusiform to hippocampal-directed connectivity in the gamma range ‘after’ surprise. No GC effects between the hippocampus and occipital cortex as a function of entropy were significant in either direction (Extended Data Fig. 10d,e). The absence of directed information flow alongside clear functional modulation (prestimulus ripple-associated reduction in occipital gamma activity) suggests that hippocampal influence on uncertainty processing either operates through state preparation rather than continuous information transfer or is mediated along the processing hierarchy to enable such state preparation. As there were few participants with contacts in both cortical regions (*n* = 4), GC between occipital and fusiform cortex was not examined. All hippocampal–cortical interactions reported above are summarized schematically for high entropy (prestimulus time periods) and high surprise (poststimulus time periods) in Fig. 6.

## Discussion

We measured hippocampal and cortical activity using depth electrodes in patients with epilepsy as they observed sequences of simple visual stimuli drawn from a block-specific distribution, yielding varying levels of stimulus-bound uncertainty and surprise. Electrophysiological intracranial activity was examined prestimulus and poststimulus to assess prediction under different levels of uncertainty and subsequent validation against observed inputs, respectively. Our results highlight an important hippocampal function in generating putative predictions and communicating them downstream to the cortex. Specifically, our findings suggest that hippocampal ripples are key to this generative process—ripple probability was highest in the prestimulus window when uncertainty was high but not at its maximum, and prestimulus ripple duration increased with entropy. These prestimulus ripples were associated with subsequent faster responses to poststimulus surprise in the fusiform cortex. This is exactly consistent with a role of hippocampal ripples reporting the predictability of an upcoming stimulus and thereby affording the ensuing prediction errors greater precision, in the context of greater expected information gain (that is, higher entropy).

The properties and distribution of ripples as a function of peri-stimulus time and entropy strongly suggest that they have an important role in generating a certain kind of prediction; namely, a prediction of predictability. Previous work in rodents has demonstrated that ripples represent future trajectories of experienced and new paths^41–43^, indicative of a generative process. Our results provide support for, and extend, these previous studies by showing increased ripple probability in uncertain contexts before stimulus onset, in awake human patients, as they anticipate upcoming sensory input. Specifically, predictive processing under uncertainty may represent an integral part of the proposed role for ripples in planning future actions^40,41^. Our findings are in keeping with a recent synthesis of rodent studies suggesting that ripple trajectories comprise not only the path to be chosen or the path just completed, but also many of the potential options available^62^. With an increasing number of options—and implicit unpredictability of ensuing outcomes—the ripple rate would be expected to increase, as observed here. This is also in line with recent fMRI work in humans showing successor-like representations in the hippocampus and visual cortex^29^. Notably, our visuomotor mapping task did not impose memory demands, as both stimulus and response mapping appeared on the screen simultaneously. The key manipulation was the underlying probability of the stimulus distribution within block, which we suggest elicited an increased need for precise predictive processing, and thus more ripple events, with increased uncertainty regarding the upcoming stimulus. This allowed us to elucidate an important functional role that ripples have in anticipation of sensory inputs and associated action planning^48^.

The increased frequency of prestimulus ripples occurred around the same time as the negative association between entropy and gamma power (overlapping in 80–97.5 Hz), namely, around 800 ms prestimulus. The early hippocampal gamma effect (that is, negative association with entropy) is—on a predictive coding narrative—consistent with an attenuation of neuronal fluctuations in populations reporting prediction errors (for example, superficial pyramidal cells) in proportion to the predicted precision of subsequent (stimulus-bound) prediction errors^11^. Such an early effect is consistent with (Bayesian belief) an updating of information from the previous trial and broadcasting the ensuing predictions down the cortical hierarchy. This finding also suggests that detected ripples were likely discrete events, dissociable from gamma activity^37,63^. The observed reduction in gamma activity (spanning frequencies lower than that of the ripple activity) occurred in the prestimulus period on trials in which prestimulus ripples were evident (Extended Data Fig. 4). This co-occurrence might be driven by the opposing effects of acetylcholine on ripple generation and gamma power (increase in ripple rate accompanied by decreased gamma-band activity, and vice versa^37^). This is interesting because acetylcholine has been implicated in the encoding of precision in predictive processing in several computational studies^9,58,64^.

To demonstrate that ripples carry predictive information that is subsequently propagated to the cortex, it is important to establish peri-ripple modulation of behavior or cortical activity, as suggested by predictive processing^5,16^. There are mixed findings in the literature, with some studies showing no ripple occurrence-related modulation of subsequent behavior in rodents^48–50^. Nevertheless, hippocampal ripples modulated cortical activity in several ways; in line with previous findings from rodents and nonhuman primates^45–47^, we found an overall increase in high gamma power in fusiform and occipital cortex around the ripple time, supporting the notion that the occurrence of ripples facilitates enhanced interaction between the hippocampus and cortex. These findings demonstrate that ripples implement general precision-weighting mechanisms that modulate cortical readiness across different computational contexts, rather than being tied to any single variable.

In addition to the cortical modulation across all ripple trials, a unique modulation of prestimulus ripples on cortical processing was observed, in keeping with their predictive role. First, we found a suppression of occipital gamma activity that was time locked to the hippocampal ripple event. Together with the overall positive association between entropy and occipital gamma power prestimulus, this suggests that the ripple-triggered suppression may reflect an overriding hippocampal signal over lower-level cortical prediction of the upcoming stimulus, or facilitate a sharper representation of the possible outcomes in occipital cortex^14,65^. We suggest that this suppression represents state optimization that prepares the cortex for more effective prediction error processing when stimuli arrive. Therefore, although there is a general increase (positive association) between occipital gamma as entropy increases, this is modulated (suppressed) in the presence of prestimulus hippocampal ripples (Extended Data Fig. 6a). This anticipatory prediction generation is exactly what predictive coding theory predicts; when upcoming stimuli are unpredictable (high entropy), the visual system must prepare more robust predictions to effectively process the expected information.

Second, the presence of prestimulus ripples modulated poststimulus fusiform activity; while there was an overall positive association between trial-wise surprise and gamma power in fusiform around 300 ms poststimulus, when splitting trials based on the presence of prestimulus hippocampal ripples, we found a faster fusiform gamma response to surprise, compared to trials without prestimulus ripples. Gamma-band activity has been shown to support bottom-up processing^35,36^, so that the faster response to surprise in the presence of prestimulus ripples indicates that a prior prediction facilitates signaling of prediction errors^31^, and their propagation of the visual processing hierarchy. Further support for this claim arises from the E/I analyses, which provided direct neurobiological evidence for our precision-weighting hypothesis—prestimulus hippocampal ripples enhance the precision afforded to subsequent prediction errors through enhanced inhibitory control mechanisms, resulting in faster, stronger and more controlled cortical responses to surprising stimuli.

Furthermore, we also observed increased fusiform to hippocampus information flow in high surprise as reflected in GC for gamma activity from 150 to 650 poststimulus, which we associate with bottom-up prediction error signaling. This is compatible with a view that prestimulus hippocampal ripples modulate (or prime) the fusiform to respond at earlier latencies to surprising stimuli (top-down effect), and the fusiform poststimulus gamma response to surprise feeds back to the hippocampus (bottom-up effect). The precise nature of hippocampal ripple-triggered changes in cortical state remains to be determined (for example, cortical layer-specific engagement of inhibitory interneurons), although the simplicity of the current behavioral task lends itself to further mechanistic interrogation using nonhuman animal models, where homologous predictive coding mechanisms are evident^66^.

Finally, we also observed poststimulus hippocampal ripples, but did not, however, find a relationship between these and behavioral measures or cortical activity. Different cortical effects linked to prestimulus versus poststimulus hippocampal ripples could imply that these ripples arise in different neuronal populations whose outputs are routed differently. Therefore, it is possible that poststimulus ripples serve a different functional role^38,67^, or facilitate communication with other brain regions, such as the prefrontal cortex^45,46^.

In conclusion, our findings speak to an important function of hippocampal ripples in predictive processing, reporting the predictability or expected information gain of stimuli (that is, prediction errors) before they are encountered, thereby facilitating their propagation through the visual cortex (in the absence of any memory demands). More specifically, our results reveal an increase in ripple events in uncertain trials, before stimulus onset, and subsequent modulation of cortical activity, pointing to enhanced hippocampal–cortical communication facilitated by ripples that serve the propagation of precise prediction errors. These findings reveal complementary mechanisms through which hippocampal ripples achieve their modulatory effects—preparing cortical states for upcoming uncertain stimuli (entropy pathway) and enhancing the precision of prediction–error responses when surprising events occur (surprise pathway).

## Methods

### Participants

Eighteen patients with medication-resistant presurgical epilepsy (mean age = 37.3 years, s.d. = 11.7; eight males) with depth electrodes surgically implanted to aid seizure focus localization took part in the experiment. Data were acquired at two epilepsy centers, one in Madrid and one in Zurich. All patients signed informed consent before participating in the experiment. Implantation sites were chosen solely on the basis of clinical criteria. Patients had normal or corrected-to-normal vision and had no history of head trauma or encephalitis. All patients had electrodes implanted in the hippocampus. For patients showing unilateral hippocampal sclerosis, only the nonpathological side was included in the analysis; in all other patients, hippocampi were radiologically normal on preoperative MRI.

Of the 18 patients who completed the task, 1 patient was excluded from any analysis due to poor performance on the task (less than 25% accuracy on all trials). We therefore analyzed electrophysiological responses from 17 patients. In total, we analyzed contacts in the right hippocampus from seven patients, and eight patients with contacts on the left side. Of these 17 patients, 8 patients also had electrodes in the occipital cortex, and 7 patients also had contacts in the fusiform. Our sample size was not determined before the experiment, but it is equal or larger than that reported in previous publications using cognitive tasks with intracranial electroencephalography (iEEG)^51,68^. This study has been approved by the local ethics committees of Hospital Ruber Internacional and Kantonale Ethikkommission (PB-2016-02055).

### Stereotactic electrode implantation

A contrast-enhanced MRI was performed pre-operatively under stereotactic conditions to map vascular structures before electrode implantation, and to calculate stereotactic coordinates for trajectories using the Neuroplan system (Integra Radionics). For patients whose data were collected in Madrid (*n* = 12), DIXI Medical Microdeep depth electrodes (multicontact, semirigid, diameter of 0.8 mm, contact length of 2 mm, intercontact isolator length of 1.5 mm) were implanted based on the stereotactic Leksell method. For patients whose data were collected in Zurich (*n* = 5), the depth electrodes (diameter of 1.3 mm, eight contacts of 1.6 mm length and spacing between contact centers of 5 mm; Ad-Tech, www.adtechmedical.com) were stereotactically implanted in the medial temporal lobes.

### Data acquisition

In Madrid, iEEG activity was acquired using an XLTEK EMU128FS amplifier (XLTEK). iEEG data were recorded at each electrode contact site at a 500 Hz sampling rate (online bandpass filter of 0.1–150 Hz) and referenced to linked mastoid electrodes. For four patients, the data were recorded with a higher sampling rate, but were later downsampled to 500 Hz. In Zurich, data were acquired using a Neuralynx ATLAS system with a sampling rate of 4000 Hz (online bandpass filter of 0.5–1000 Hz) against a common intracranial reference, and then downsampled to 500 Hz. Data from the two centers were comparable, and we did not observe differences across centers in any of the analyses reported in the Results section.

### Electrode localization: hippocampus

To localize electrodes with contacts in the hippocampus, we used the manual procedure described previously^68^. For each patient, the postelectrode placement CT (post-CT) was coregistered to the preoperative T1-weighted MRI (pre-MRI). To optimize coregistration, both brain images were first skull stripped. For CTs, this was done by filtering out all voxels with signal intensities between 100 and 1,300 HU. Skull stripping of the pre-MRI proceeded by first spatially normalizing the image to MNI space using the New Segment algorithm in SPM8 (http://www.fil.ion.ucl.ac.uk/spm). The resultant inverse normalization parameters were then applied to the brain mask from SPM8 to transform the brain mask into the native space of the pre-MRI. All voxels in the pre-MRI that were outside the brain mask and with a signal value in the top 15% were filtered out. The skull-stripped pre-MRI was then coregistered and resliced to the skull-stripped post-CT. Next, the pre-MRI was affine registered to the post-CT, thus transforming the pre-MRI image into a native post-CT space. The two images were then overlaid, with the post-CT thresholded such that only electrode contacts were visible. For all patients, only contacts in the hippocampus head and body were selected. For patients with multiple electrodes localized in the hippocampus, only the most anterior contacts were used in the analyses (Fig. 1c). Electrode contacts for each patient are shown in Supplementary Fig. 1.

### Electrode localization: cortical regions

To localize electrodes with contacts in the occipital and fusiform cortices, we used a semiautomatic procedure with Lead-DBS^69^ (https://www.lead-dbs.org/). First, the postoperative CT was registered to the preoperative MRI using a two-stage linear registration (rigid followed by affine) as implemented in Advanced Normalization Tools^70^. The images were then normalized to the MNI template based on the pre-MRI using the SyN registration approach as implemented in Advanced Normalization Tools. To reduce bias introduced by brain shift, the brain shift correction implemented in Lead-DBS was performed on the postoperative CT. Manual prereconstruction was then performed, in which the tip of each electrode and another point along its trajectory were manually marked, followed by automatic reconstruction guided by the electrode specification (that is, the number of contacts and their spacing). The reconstructed electrodes were then visually inspected and refined, and the processes were iterated in case of any misalignments. Once all electrodes (for any given patient) have been reconstructed, they were visualized using Lead-Group^71^. After this process, each electrode contact was associated with MNI coordinates. To identify contacts in our cortical regions of interest, a custom MATLAB code, together with the FindStructure function (https://alivelearn.net/?p=1456), was used. Each MNI coordinate was associated with a label from the Automated Anatomical Labeling atlas. For occipital cortex, MNI coordinates labeled as ‘inferior occipital’ or ‘middle occipital’ were used; for fusiform, the ‘fusiform’ label was selected. The selected contacts were then visually inspected with the Automated Anatomical Labeling overlay in MRIcron, as well as in native space (Fig. 1d and Supplementary Fig. 1).

### Behavioral task

Patients performed a visuomotor mapping task, consisting of 12 blocks with 40 trials per block, using the same design as discussed in ref. ^20^. Each trial included a brief presentation of a colored shape, for 500 ms, with an interstimulus interval of 2200 ms. In all trials within a block, two colors and two shapes were combined to form four possible outcomes, with different stimuli presented in the different blocks. Patients were asked to respond to the sampled item by pressing a key to identify the target’s position in the row (Fig. 1a). Each trial used an independent sample from a distribution that remained constant within a block, but that varied over blocks. There was no underlying sequence governing stimulus presentation; only the relative proportions of stimuli were varied from block to block. Two information theoretic measures, entropy and surprise, were then calculated (Fig. 1b). Surprise quantifies the improbability of a given event:

$$I\left({x}_{i}\right)=-\mathrm{ln}\,P\left({x}_{i}\right)$$

Entropy quantifies the expected (running average of) surprise overall the trials:

$$H(X)=\sum -P\left({x}_{i}\right)\mathrm{ln}\,P\left({x}_{i}\right)$$

Patients were told the proportion of colored shapes presented within block was random, and independent of the other blocks in the task. To account for nonspecific time effects within block, the mean entropy overall blocks was calculated for each patient. Trial number is therefore accounted for by the inclusion of mean entropy values that are the same across blocks. Therefore, for each trial, three values of interest were computed—entropy, surprise and mean entropy. These values were modeled from the perspective of an ideal Bayesian observer, using the Dirichlet distribution^20^. Only correct responses given within 1 s of stimulus onset were used for subsequent analyses.

### Electrophysiological data analysis

#### Preprocessing

iEEG data analysis was carried out using the FieldTrip toolbox^72^ (https://www.fieldtriptoolbox.org; v20200310) running on MATLAB R2019b (MathWorks). For all patients and regions of interest, recordings were transformed to a bipolar derivation by subtracting signal from adjacent electrode contacts within the region of interest (hippocampus, occipital cortex and fusiform). Previous studies demonstrate that bipolar referencing optimizes estimates of local activity^73,74^ and connectivity patterns across brain regions^75^, as well as for analysis of SWRs^52^. Data were epoched from −1 to 1 s with respect to stimulus onset (a time window selected a priori), demeaned and detrended. For each region of interest, every epoch was visually inspected for artifacts caused by epileptic spikes or electrical noise, first in the time domain and then in the TF domain. Trials with artifacts were excluded from all subsequent analyses. Of note, for analyses involving more than one region, only trials that were artifact free in all regions were used.

### SWR analyses

Ripples were detected using a previously established method^52^ (Supplementary Fig. 2a). After trial-wise artifact rejection and basic preprocessing (described above), the iEEG signal was bandpass filtered between 80 and 120 Hz using a second-order Butterworth filter. A Hilbert transform was then applied to the filtered signal to extract its instantaneous amplitude. Ripples were identified as events with a maximum amplitude three s.d. above the mean, and with a duration of at least 25 ms. Ripples that were detected within 15 ms of each other were merged. The start, peak and end time of each ripple were noted. To ensure that artifacts or interictal epileptiform discharges (IEDs; Extended Data Fig. 3) were not mistakenly classified as ripples, we used an automated procedure as described in ref. ^52^ to identify and reject IEDs. The iEEG signal was high-pass filtered at 200 Hz and a *z* score was calculated based on the gradient and amplitude of the filtered signal. Any time-point exceeding a *z* score of 5 was marked as an IED, together with the 100 ms before and after the event. All IEDs were excluded from the analysis, increasing the likelihood of the detected ripples being physiological^52^. We have also applied another ripple detection method^51^ and replicated our findings (Extended Data Figs. 1, 7 and 8). For patients who had more than one anterior hippocampal electrode (for example, head and body), we examined whether the same ripple was picked up by the different electrodes (within 20 ms). In line with previous findings in rodents^76^, we found the vast majority of ripples (96% on average) were not detected in the adjacent electrode along the hippocampal long axis.

We then examined whether ripple occurrence was influenced by peri-stimulus time and information-theoretic measures. To do so, a 2D histogram of ripple peak count using peri-stimulus time and entropy or surprise was derived for each patient. We normalized each histogram by the total number of trials per entropy/surprise bin to account for potential imbalances. This was done by calculating the 2D count histogram (time and entropy or surprise) and by multiplying by the frequency of observed ripples given available trials in each entropy or surprise bin (that is, postcleaning). The normalized histograms were then averaged across participants and tested against a uniform distribution using a Kolmogorov–Smirnov test. To identify the bins with the highest ripple counts, we also performed a mixed-effects gamma regression on the normalized ripple counts (as they follow a right-skewed distribution) with entropy and ripple peak time bin as predictors. This was done using the lme4 package^77^ in R (https://www.r-project.org/), and the following model setup: normalised_count ~ entropy × peak_time + (1|subI), family = Gamma. After the significant interaction, the top five bins with the highest estimated marginal means were identified using the pairwise function from the emmeans package (https://rvlenth.github.io/emmeans/). Finally, TFCE was calculated on the normalized 2D histogram, with a *t* test against the average ripple rate per patient. Therefore, a positive value indicates a higher-than-average ripple count observed.

Finally, to test whether the increased ripple frequency was related to entropy, over the effect of surprise, we used a standard summary statistic approach to mixed-effects modeling, using a separate Poisson GLM for each participant, for each time bin, that included a constant term, modeling the ripple rate for that participant and time bin. The predictors in this model were entropy and surprise, as well as mean entropy as covariate of no interest (as was done in all other TF analyses described below). The ensuing parameter estimates were then subject to a second-level analysis using cluster-based permutation tests as implemented in FieldTrip (using the TFCE method did not change the results).

Next, we examined ripple duration, calculated as ripple end point minus ripple start point (both determined in the detection algorithm as two *z* scores above average) as a function of peri-stimulus time and entropy and surprise. This was done using generalized linear mixed-effects models implemented by the lme4 package^77^ in R (https://www.r-project.org/). Because ripple duration follows a right-skewed distribution, we used a model from the gamma family with a random intercept for patient using the following syntax:

model ← glmer(rip_duration ~ entropy × peri_stim_time + surprise × peri_stim_time + mean_entropy + (1 | patient), family = Gamma).

A similar approach using mixed-effects linear regression was used to examine RT, which was normalized using log transformation. This approach accommodates any individual differences among participants (for example, the overall ripple rate for each participant).

### TF analyses

Time-resolved spectral decomposition was computed for each trial using seven Slepian multitapers for high frequencies (≥35 Hz) and a single Hann taper for low frequencies (<3 5Hz). The selected Slepian tapers for the analysis of high frequencies were based on windows with width of 0.4 s and a 10 Hz frequency smoothing. The time-resolved spectral estimation was done in steps of 2.5 Hz. No baseline correction was performed, given our interest in both prestimulus and poststimulus activities. Trial-wise TF estimates were then entered into a GLM; two predictors of interest (entropy and surprise) and a covariate (mean entropy) were used to predict power at each TF point. The parameter estimates for entropy, mean entropy and surprise are proportional to the range these values can assume. This resulted in a ‘first-level’ beta map (with size time × frequency) per predictor. The spectral activity was then averaged over bipolar channels, within each region, for each patient (in some cases, there was only one bipolar channel in each brain region). These beta maps were then used for statistical inference with a cluster-based permutation test with the maximum number of permutations allowed by our sample size, up to a maximum of 5,000 permutations. In each permutation step, clusters were formed by temporal and frequency adjacency using a cluster-forming threshold of *P* = 0.05. In each permutation step, a one-sample two-tailed *t* test against a value of 0 (equivalent to H0—β = 0), using a threshold of *P* = 0.025, was calculated for each TF estimate, separately for low (2.5–32.5 Hz) and high (35–160 Hz) frequencies, as well as prestimulus (−1 to 0 s) and poststimulus (0 to 1 s) time windows. Gamma frequency band is used to refer to frequencies of 30–100 Hz, whereas high gamma refers to effects above 100 Hz. The precise frequency ranges (and associated time windows) that we report for each significant effect are derived from a cluster-based permutation correction for multiple comparisons.

### Ripple-modulated cortical activity

To examine the temporal relationship between hippocampal ripples and cortical responses, we extracted TF estimates in occipital cortex and fusiform with respect to peri-ripple peak time (−0.2 to 0.2 s, selected a priori), as described above for peri-stimulus responses, for high and low frequencies. Due to the shorter time window, the selected Slepian tapers for the analysis of high frequencies were based on windows with width of 0.2 s and a 10 Hz frequency smoothing. The time-resolved spectral estimation was done in steps of 5 Hz. These TF estimates were baseline corrected by calculating the relative change with respect to a baseline period of −1.4 to −1 s peri-stimulus. Averaged TF estimates across patients were used for statistical inference using cluster-based permutations, as described above. In each permutation step, a one-sample two-tailed paired *t* test was performed between prestimulus and poststimulus periods (threshold of *P* = 0.025). Next, to examine whether prestimulus hippocampal ripples modulated poststimulus cortical responses to surprise, we used the same GLM approach as described above, fitting a trial-wise regression at each TF point with entropy, surprise and mean entropy as predictors (TF analyses).

### E/I balance analysis

E/I balance analysis of activity in fusiform cortex was performed using the ‘fitting oscillations and one over f’ (FOOOF)^61^ method as implemented in FieldTrip. To achieve this, we extracted the aperiodic exponent value for trials with or without prestimulus hippocampal ripples. The time series was first epoched from −750 to +250 ms around stimulus onset, as this reflects the majority of hippocampal ripples detected (Fig. 2c), and around the onset of the fusiform gamma response to surprise (Fig. 5b). The data were then bandpass filtered between 30 and 70 Hz, filtering out the 50 Hz line noise. Fractal spectral estimation was performed with a spectral smoothing of 2 Hz. This resulted in a single exponent value for trials with or without prestimulus ripples, for each patient. At the group level, a paired two-tailed *t* test was performed to compare the exponent values, with smaller exponents reflecting flatter spectral slopes, indicating a shift toward excitation (E>>I).

### GC analyses

Finally, to evaluate the direction of information flow between the hippocampus and our cortical regions of interest, we calculated spectral nonparametric GC as a measure of directed functional connectivity using the FieldTrip toolbox, on 500 ms time windows centered on the induced responses observed to surprise in the fusiform gyrus and hippocampus, and the induced responses to entropy in the occipital cortex and hippocampus. For patients with multiple bipolar channels in any of the regions, the most medial and anterior channel was used to compute GC. Briefly, time-resolved spectral decomposition was computed using nine Slepian multitapers (frequency range of 2–80 Hz and 10 Hz smoothing). The spectral transfer matrix was then obtained from the Fourier transformation of the data and together with the noise covariance matrix, they were used to calculate the total and intrinsic power through which GC is computed. The resulting GC values were then log-transformed to normalize the data for statistical inference. We then compared information in high versus low entropy and surprise (using a median split) in each direction (hippocampus → cortex and cortex → hippocampus) with nonparametric cluster-based permutations (using a dependent samples of two-tailed *t* test), using the maximum number of permutations available for each pair of regions.

### Reporting summary

Further information on research design is available in the Nature Portfolio Reporting Summary linked to this article.

## Online content

Any methods, additional references, Nature Portfolio reporting summaries, source data, extended data, supplementary information, acknowledgements, peer review information; details of author contributions and competing interests; and statements of data and code availability are available at 10.1038/s41593-026-02345-6.

## Supplementary information

Supplementary Information Supplementary Figs. 1–21, Supplementary Tables 1–3 and Supplementary Note (Relationship between entropy and surprise and Independent data from oddball task).

Reporting Summary

Peer Review File

## Data Availability

Preprocessed data needed to generate the figures, and over which statistics were computed, are available in the following GitHub repository: https://github.com/frdarya/GenerativeRipples.

## Code availability

Analysis codes are available in the following GitHub repository: https://github.com/frdarya/GenerativeRipples.
