# Supplementary material for: Human hippocampal ripples tune cortical responses based on predicted uncertainty
Source: Nat Neurosci. 2026 Jun 25;29(8):1987–98. doi: 10.1038/s41593-026-02345-6 (PMC13433285; doi:10.1038/s41593-026-02345-6)
Supplement: Supplementary file 1 [file 41593_2026_2345_MOESM1_ESM.pdf]

nature neuroscience

Supplementary information

https://doi.org/10.1038/s41593-026-02345-6

Human hippocampal ripples tune cortical responses based on predicted uncertainty

In the format provided by the authors and unedited

Table of Contents

Table S1 | Patient demographic and clinical information……………………………….……2

Figure S1 | Electrode localisation………………………………………………………….3

Relationship between entropy and surprise………………………………………………..4

Independent data from oddball task………………………………………………………..5

Figure S3 | Raw spectrograms across all trials in each of the region of interest…………….…….7

Figures S4-S18 | Averaged, and example single trial, ripple traces from hippocampal and adjacent contacts for each patient.……………………………..……..…………………8-17

Figure S19 | Ripple peak frequency per patient…….……...………………………………...18

Figure S20 | Mean IEDs per patient…………………………………………………………19

Figure S21| Example IEDs per patient……………………………………………………….…20

Table S1 | Patient demographic and clinical information. M/F male, female: L/R left, right

**ID**	**age**	**gender**	**Lesion location**
**3**	55	M	Right temporal neocortex
**6**	48	M	Left hippocampal sclerosis
**8**	28	F	Extensive right posterior dysplasia involving the convexity and medial aspect of parietal, occipital and posterior temporal lobes
**9**	38	M	Right temporal lobe
**13**	42	F	Right basal temporal cortex
**15**	35	F	Left temporal pole
**16**	30	M	Medial wall of the left parietal region (precuneus and posterior cingulum)
**25**	29	M	Right posterior temporobasal region
**31**	48	F	Left frontal or fronto-temporal
**32**	59	M	Bilateral temporal pole (crisis registered only from the left side)
**36**	28	F	
**37**	24	F	Non-lesional
**6z**	29	F	Unclear
**8z**	30	F	Non-lesional
**10z**	56	M	Right hippocampus
**11z**	32	M	Occipital lesion
**12z**	24	F	Right hippocampus

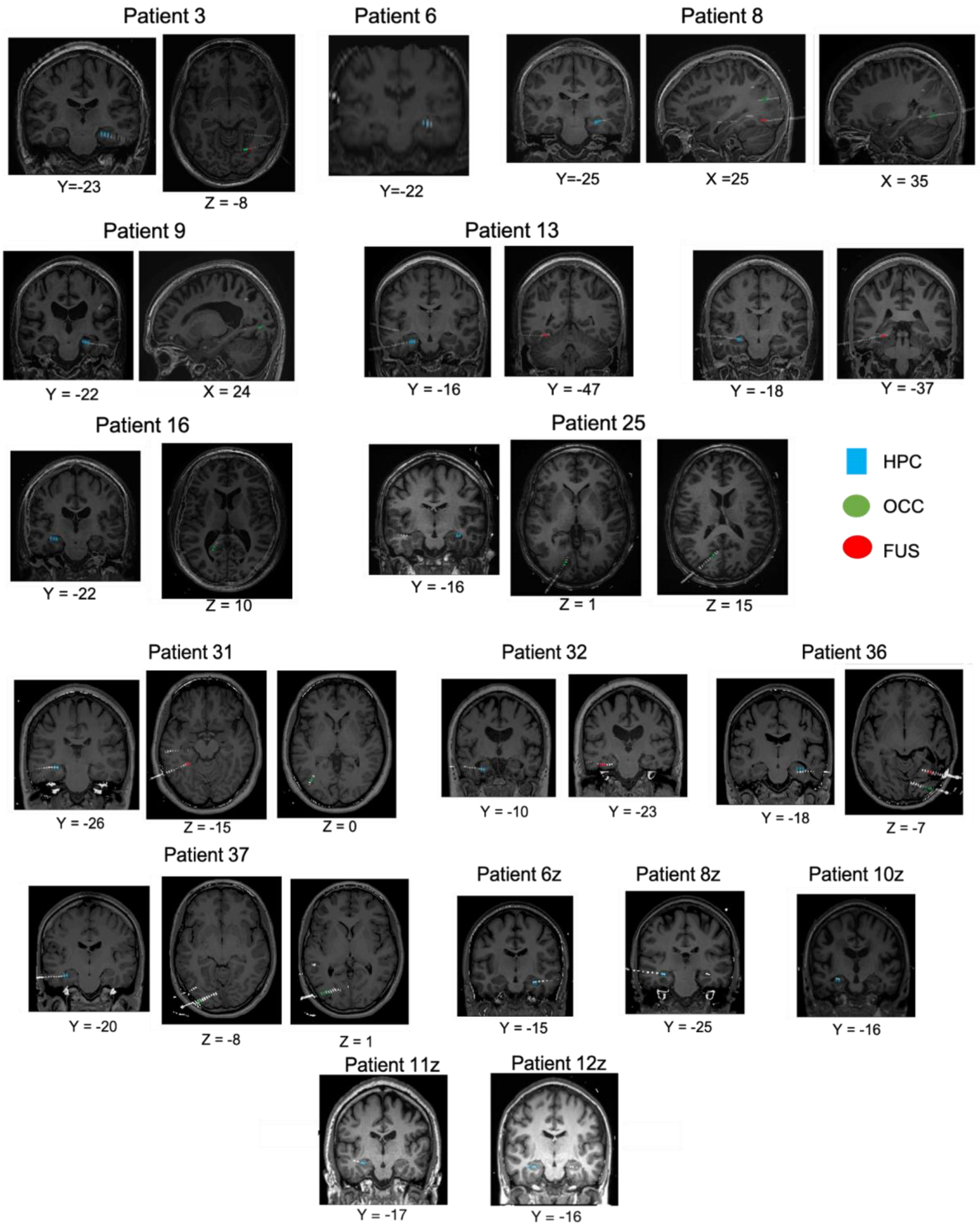

Figure S1 Contact localization | Patient-specific contacts in the hippocampus (HPC; yellow), occipital cortex (green; OCC) and fusiform (red; FUS). For all patients, post-operative CT images from each patient have been normalised and co-registered with their corresponding pre-operative MRI scans in MNI space and superimposed to display hippocampal, fusiform and occipital cortex contacts (CTs have been thresholded so as to only show electrode contacts).

Relationship between entropy and surprise

Whilst we observed a linear correlation between entropy and surprise ($r = 0.3$, $p < 0.001$), a linear function does not provide the best explanation of this relationship. A quadratic model ($x = a + \beta_1 y + \beta_2 y^2$) had a significantly larger explained variance (adjusted R^2) ($t(14) = 13.06$, $p < 0.001$, Cohen's $d = 2.1$), and smaller Akaike information criterion (AIC)($t(14) = 5.75$, $p < 0.001$, Cohen's $d = 1.48$), indicating that it provides a better fit of the data than a simple linear model (here the comparison was against a linear model of $x = a + \beta y$, as it is not possible to fit a function to a sideways parabola like the one presented in Figure 1b, because there will be multiple y values for each x, hence the transformation of y and x in this case). We also compared the equivalent of the quadratic model without flipping the axis (predicting surprise using entropy; $y = \sqrt{x}$, which also provided a better fit than the linear model [adjusted R^2: $t(14) = 6.1$, $p < 0.001$, Cohen's $d = 1.56$; and marginally using AIC: $t(14) = 1.47$, $p = 0.16$, Cohen's $d = 0.37$]. Finally, the exponential model was also significantly better than the linear one [adjusted R^2: $t(14) = 7.56$ $p < 0.001$, Cohen's $d = 0.98$; AIC: $t(14) = 5.83$, $p < 0.001$, Cohen's $d = 1.95$.

Independent data from oddball task

To further demonstrate that hippocampal ripple rate does not increase with surprise, we next analysed data from an independent control experiment employing a classical "oddball" paradigm[56] . Patients were presented with word lists, with infrequent oddball stimuli (either perceptually distinct or negatively valenced nouns) appearing amongst standard control nouns in the same sequential position within each list. This paradigm did not include an entropy manipulation: all nouns were presented only once and were thus equally unpredictable. Oddball stimuli are surprising by design (i.e., this is standard paradigm for eliciting prediction errors), yet we found no increased ripple count for oddball stimuli compared to control words.

In brief, patients undergoing hippocampal intracranial recordings at the Ruber International Hospital, (Madrid, Spain) performed this visual oddball task (for full details, see [56]). Patients were presented with lists of 14 Spanish nouns. Nouns were presented visually at a rate of one every 3 s (stimulus duration, 1 s), centrally in black font on a white background. Patients were required to make a push-button response to indicate whether the first letter in each noun contained an enclosed space (shallow encoding task). All 14 nouns in each list were of the

same semantic category. Of these, 12 nouns were emotionally neutral and presented in the same font (standard nouns). The first five nouns in each list were always standard nouns to set the context. A perceptual oddball was presented in a different font but was emotionally neutral (1/14 = 7.14% probability of occurrence). An emotional oddball was aversive in content but perceptually identical to neutral nouns. Out of the 12 standard nouns in each list, two were selected as control nouns to be used in electrophysiological analyses. Like the oddballs, these control nouns could not occur within the first five words in a list. Moreover, like oddballs, they could not be presented immediately before or after an oddball, to control for potential carryover effects of oddballs. 6 s after the presentation of the 14th word of each list, a number was presented indicating the onset of a 30 s distraction task during which patients were instructed to count backwards in threes (out loud) from the presented number. The distractor task was followed immediately by instructions to freely recall the words presented in the preceding list. The entire procedure was repeated for the 30 lists. Three patients did not complete the full experiment (completing 22, 22 and 19 lists respectively).

A total of 14 patients with hippocampal electrode contacts were included in these analyses (Table S2): 6 left, 6 right and one bilateral hippocampal electrodes (for which the ripples were detected separately for the left and the right electrode and then averaged over the two electrodes).

Hippocampal iEEG data were preprocessed identically to the main experiment manipulating both entropy and surprise. The Vaz method was applied for ripple detection. Ripple counts for each trial type (oddballs and control nouns) were binned in 250 ms windows from -500 ms pre-stimulus to +1500 ms, and averaged over all lists performed by each patient.

Average peri-stimulus ripple counts perceptual oddballs and control nouns for each time bin, averaged over patients is shown in Figure S1. We focus on perceptual oddballs vs control words here, as this is the classical task. We performed a Condition (Perceptual oddball vs Control words) x Time window (8 time windows in steps of 250ms between -500ms and 1500ms) repeated measures ANOVA to compare the ripple rate between conditions over time. There was no main effect of Condition ($F(1,12) = .043$, $p = .840$, $\eta^2_p = .004$), no main effect of time window ($F(3.60,43.16) = 2.11$, $p = .102$, $\eta^2_p = .15$) and – critically – no significant Condition x Time window interaction ($F(4,48.05) = 1.71$, $p = .163$, $\eta^2_p = .12$). The ANOVA results thus demonstrated no reliable difference in ripple rate between perceptual oddballs and control words, which we further highlight by performing post-hoc paired t-tests between conditions for all time windows. No time window showed a significant difference

when correcting for multiple comparisons (see table S3 for t-values and fdr corrected p-values). Ripple responses to emotional nouns also failed to show any significant difference to control nouns, and will be reported further elsewhere.

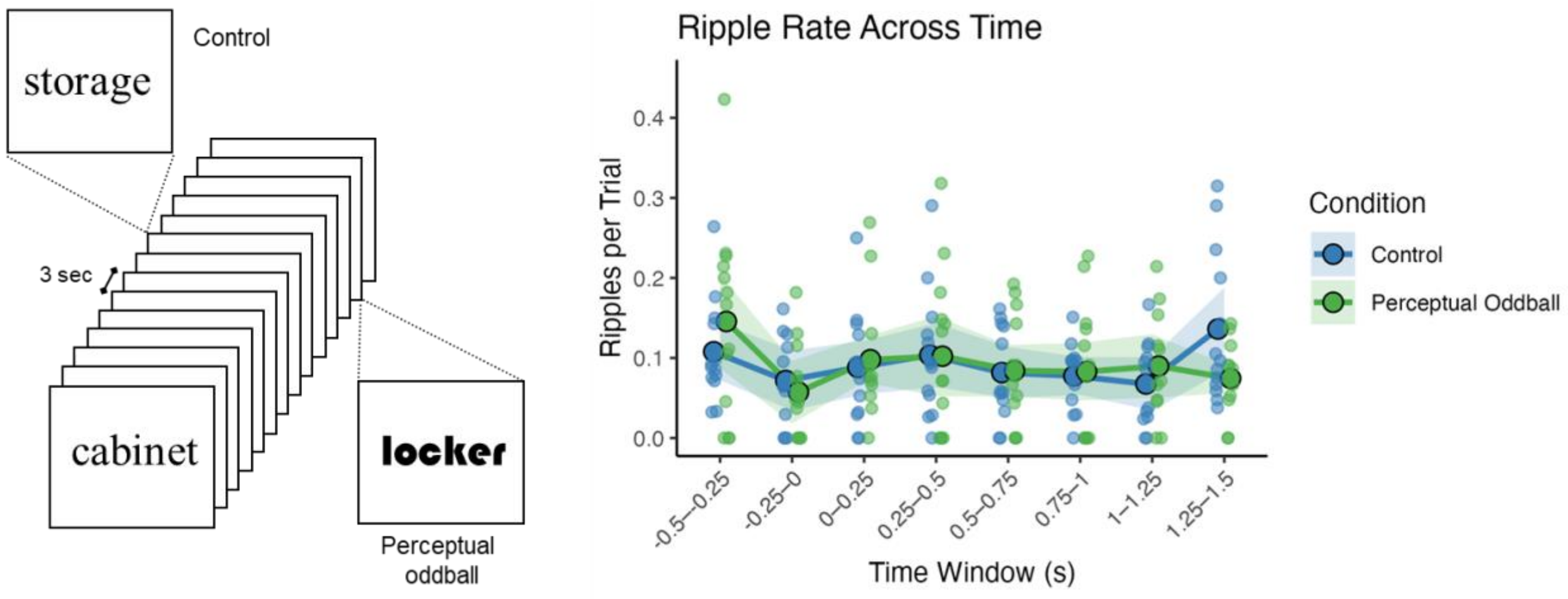

Figure S2 | Hippocampal ripple rate is not increased by surprising, infrequent perceptual "oddballs". *Left*: Experimental paradigm. Insets depict an example perceptual oddball ("locker") and control noun ("storage"). *Right*: Peri-stimulus ripple rate is plotted in 250ms bins, collapsed over trials within-patient (individual data points) and averaged over patients (larger circles). Perceptual oddballs (green) do not evoke increased ripple activity compared to control nouns (blue). Shaded bars depict 95% confidence intervals.

Table S2 | Patient information for participants in verbal oddball task. Demographic and clinical details are provided for those patients who did not perform the main task. Abbreviations: M = Male, F = Female, R = right, L = left, B = Bilateral.

Patient ID	Hippocampal Hemisphere	Number of lists completed	Age	Gender	Lesion Location
3	R	30			
8	R	30			
10	R	30	59	M	Right anterior temporal pole and basal area
15	L	22			
16	B	30			
21	L	30	31	F	Left occipital horn heterotopia
25	L	30			
27	L	30	25	M	Left temporal pole
28	L	30	25	M	Left temporal lateral region
32	L	19			
33	R	22	22	F	Right parietal cortex
36	R	30			
38	R	30	18	M	Right temporal lateral region

Table S3 | Hippocampal ripple rate is not significantly increased by surprising, infrequent perceptual "oddballs" relative to standard control nouns in any peri-stimulus time window tested. In the longest latency time window post-stimulus, there were significantly more ripples following control nouns than perceptual oddballs, but this did not survive correction for multiple comparisons.

**Time windows**	***t(12)***	***p value (uncorrected)***	***p value* (fdr corrected)**	***Cohen's d***
-500ms to -250ms	1.87	.087	.347	.41
-250ms to 0ms	-.62	.550	.981	.23
0ms to 250ms	.49	.634	.981	.16
250ms to 500ms	.02	.983	.983	.01
500ms to 750ms	.17	.865	.983	.07
750ms to 1000ms	.35	.735	.981	.11
1000ms to 1250ms	1.25	.236	.628	.42
1250ms to 1500ms	-2.58	.024	.194	.81

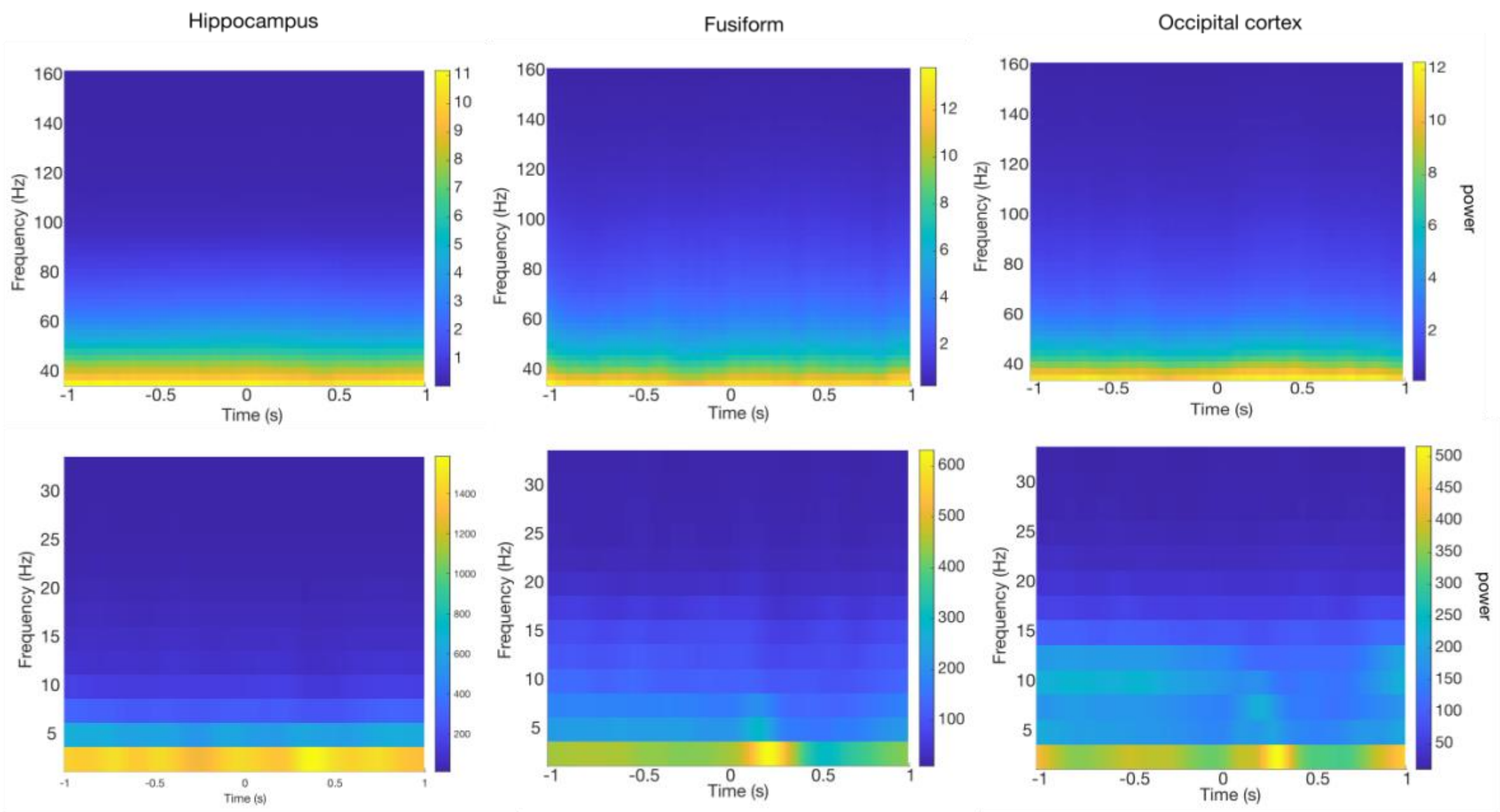

Figure S3 | Raw spectrograms across all trials in each of the region of interest.

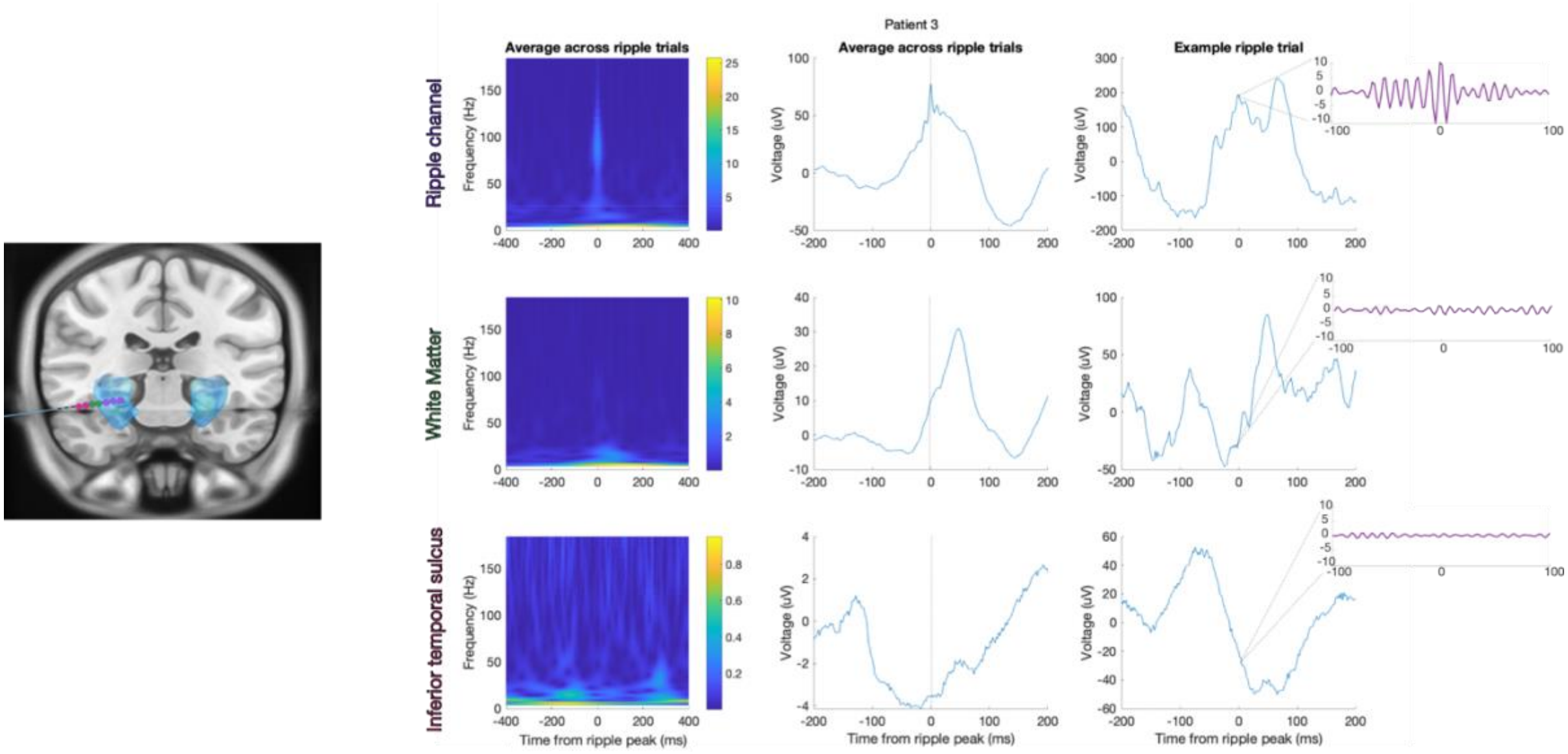

Figure S4 | Averaged, and example single trial, ripple traces from hippocampal and adjacent contacts for Patient 3. *Left.* Electrode localisation shown on the smoothed hippocampus mask from the Automated Anatomical Labelling atlas for visualization; blue) illustrates the contacts (coloured circles) from which data are presented in the panels on the right. Purple circles: 3 hippocampal contacts i.e., two bipolar channels. Green circles: the two contacts adjacent to the hippocampus are in white matter (medial contact)/inferior temporal sulcus (lateral contact). Red circles: the two subsequent contacts are in inferior temporal sulcus (medial contact)/white matter (lateral contact). *Right.* Top, bottom and middle rows: ripple-locked activity from the hippocampal (Ripple) channels (purple circles), and adjacent bipolar channels "White matter" (corresponding to green circles) and "inferior temporal sulcus" (corresponding to red circles) . The left and centre columns show the average peri-ripple wavelet spectrogram across all ripple trials (n = 320), and average raw field potential centred on ripple peak, respectively. The right column shows an example ripple trial (raw field potential centred on ripple peak) with an inset showing the ripple band activity (band pass filtered at 80-120Hz).

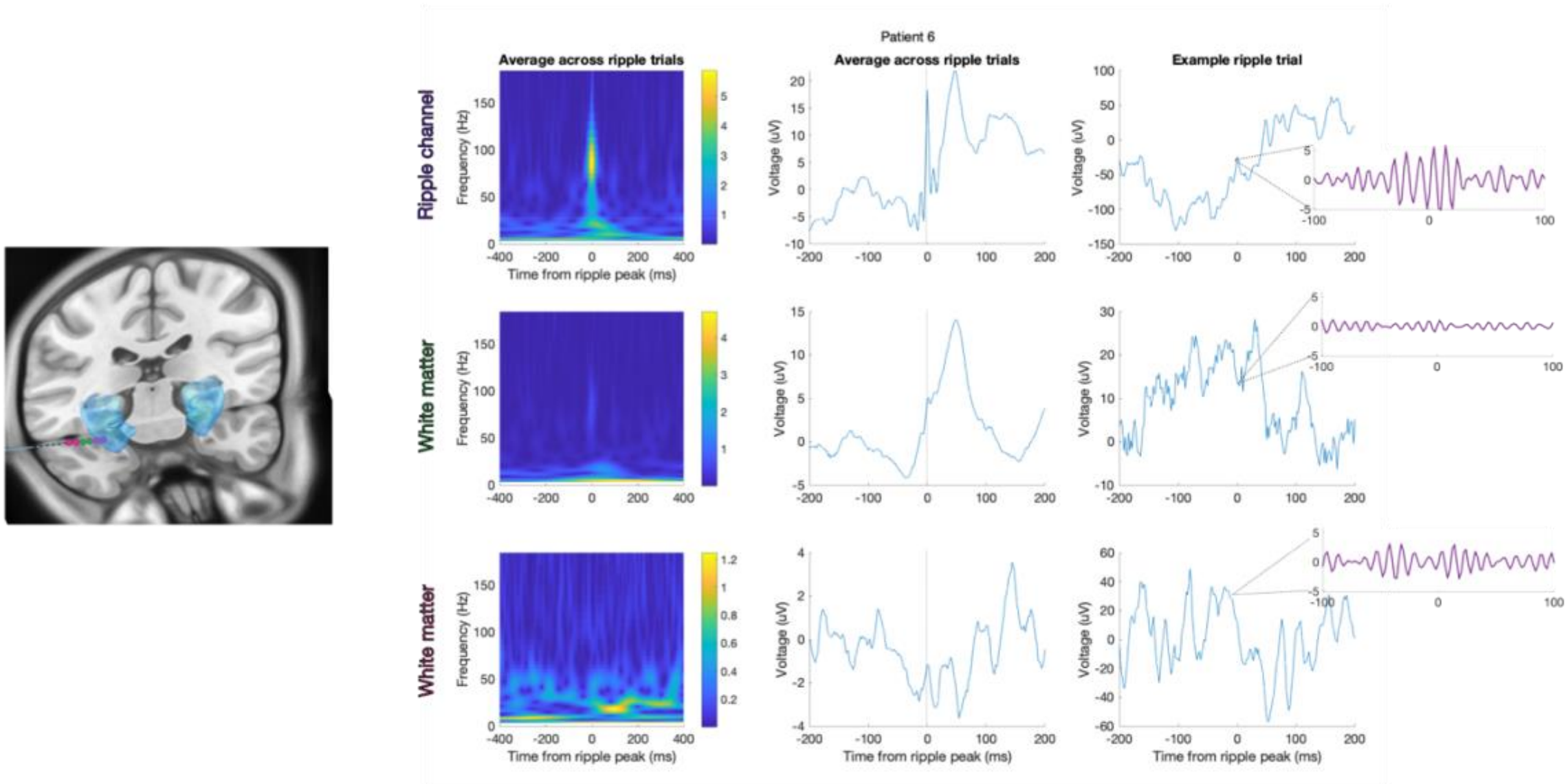

Figure S5 | Averaged, and example single trial, ripple traces from hippocampal and adjacent contacts for Patient 6. *Left*. Electrode localisation shown on the smoothed hippocampus mask from the Automated Anatomical Labelling atlas for visualization; blue) illustrates the contacts (coloured circles) from which data are presented in the panels on the right. Purple circles: 2 hippocampal contacts i.e., one bipolar channel. Green circles: the two contacts adjacent to the hippocampus are in white matter. Red circles: the two subsequent contacts are in white matter. *Right*. Top, bottom and middle rows: ripple-locked activity from the hippocampal (Ripple) channel (purple circles), and adjacent bipolar channels “White matter” (corresponding to green circles) and “White matter” (corresponding to red circles) . The left and centre columns show the average peri-ripple wavelet spectrogram across all ripple trials (n = 160), and average raw field potential centred on ripple peak, respectively. The right column shows an example ripple trial (raw field potential centred on ripple peak) with an inset showing the ripple band activity (band pass filtered at 80-120Hz).

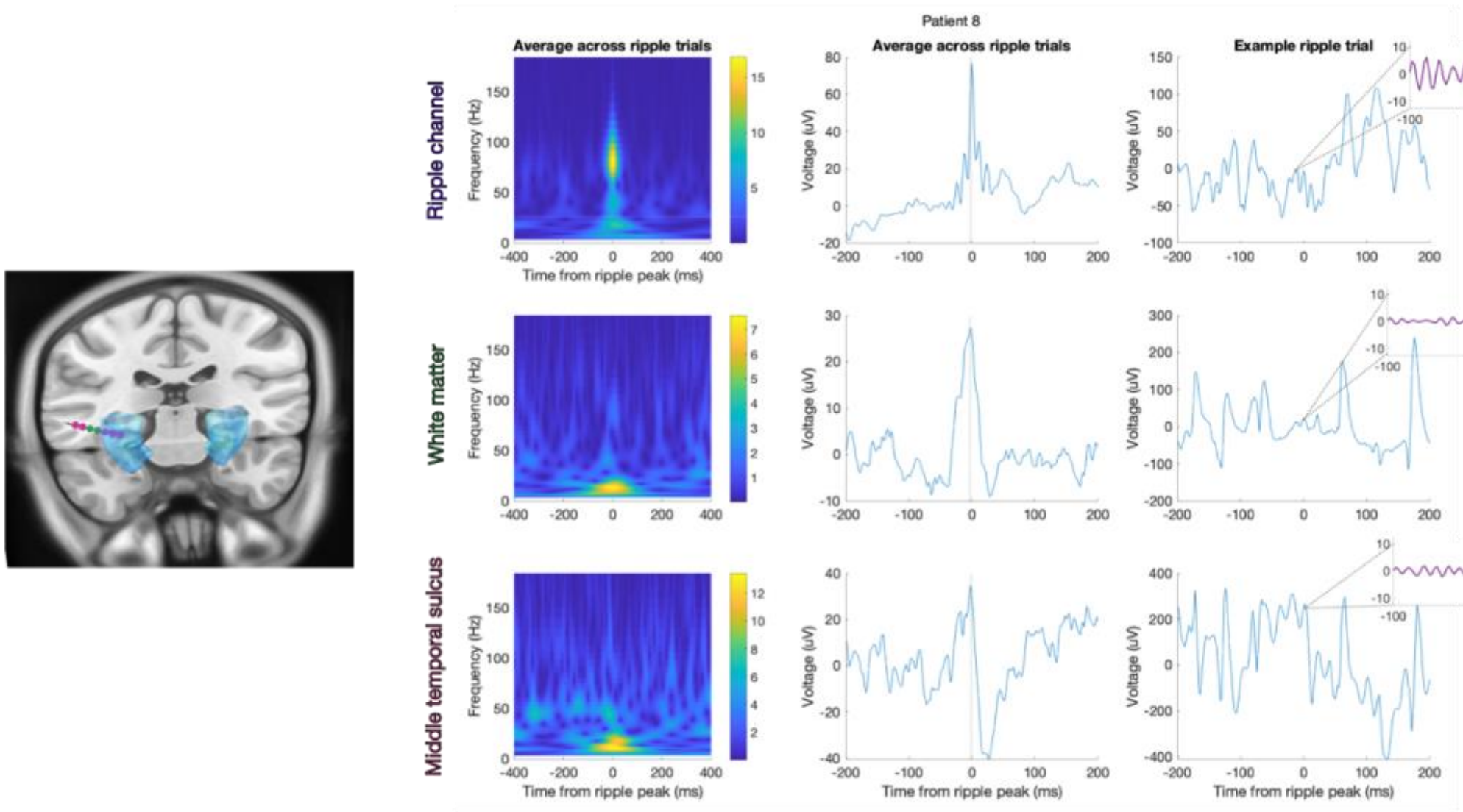

Figure S6 | Averaged, and example single trial, ripple traces from hippocampal and adjacent contacts for Patient 8. *Left*. Electrode localisation shown on the smoothed hippocampus mask from the Automated Anatomical Labelling atlas for visualization; blue) illustrates the contacts (coloured circles) from which data are presented in the panels on the right. Purple circles: 3 hippocampal contacts i.e., two bipolar channels. Green circles: the two contacts adjacent to the hippocampus are in white matter. Red circles: the two subsequent contacts are in middle temporal sulcus. *Right*. Top, bottom and middle rows: ripple-locked activity from the hippocampal (Ripple) channels (purple circles), and adjacent bipolar channels “White matter” (corresponding to green circles) and “inferior temporal sulcus” (corresponding to red circles). The left and centre columns show the average peri-ripple wavelet spectrogram across all ripple trials (n = 210), and average raw field potential centred on ripple peak, respectively. The right column shows an example ripple trial (raw field potential centred on

ripple peak) with an inset showing the ripple band activity (band pass filtered at 80-120Hz).

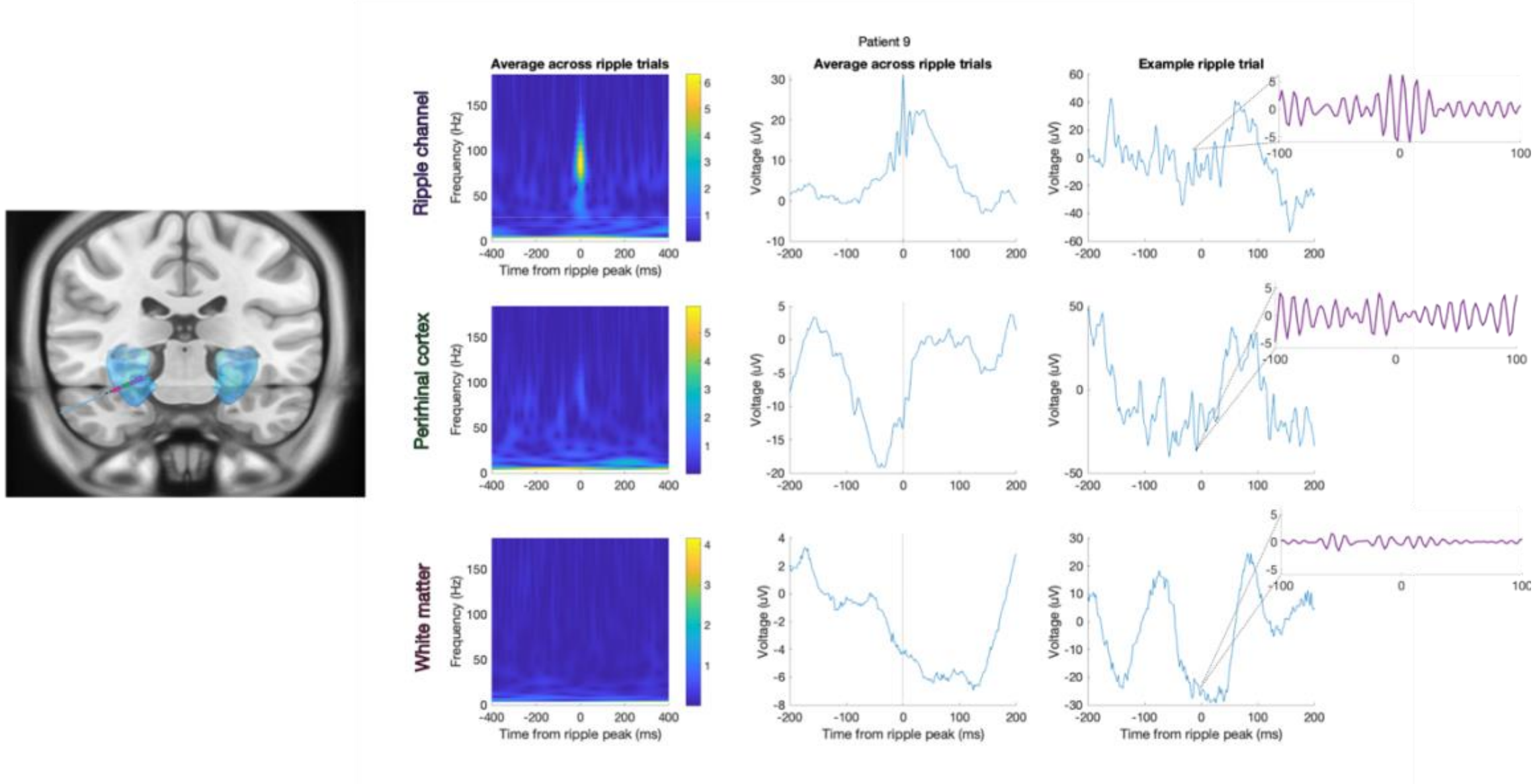

Figure S7 | Averaged, and example single trial, ripple traces from hippocampal and adjacent contacts for Patient 9. *Left*. Electrode localisation shown on the smoothed hippocampus mask from the Automated Anatomical Labelling atlas for visualization; blue) illustrates the contacts (coloured circles) from which data are presented in the panels on the right. Purple circles: 3 hippocampal contacts i.e., two bipolar channels. Green circles: the two contacts adjacent to the hippocampus are in perirhinal cortex. Red circles: the two subsequent contacts are in white matter. *Right*. Top, bottom and middle rows: ripple-locked activity from the hippocampal (Ripple) channels (purple circles), and adjacent bipolar channels "Perirhinal cortex" (corresponding to green circles) and "White matter" (corresponding to red circles). The left and centre columns show the average peri-ripple wavelet spectrogram across all ripple trials (n = 160), and average raw field potential centred on ripple peak, respectively. The right column shows an example ripple trial (raw field potential centred on ripple peak) with an inset showing the ripple band activity (band pass filtered at 80-120Hz).

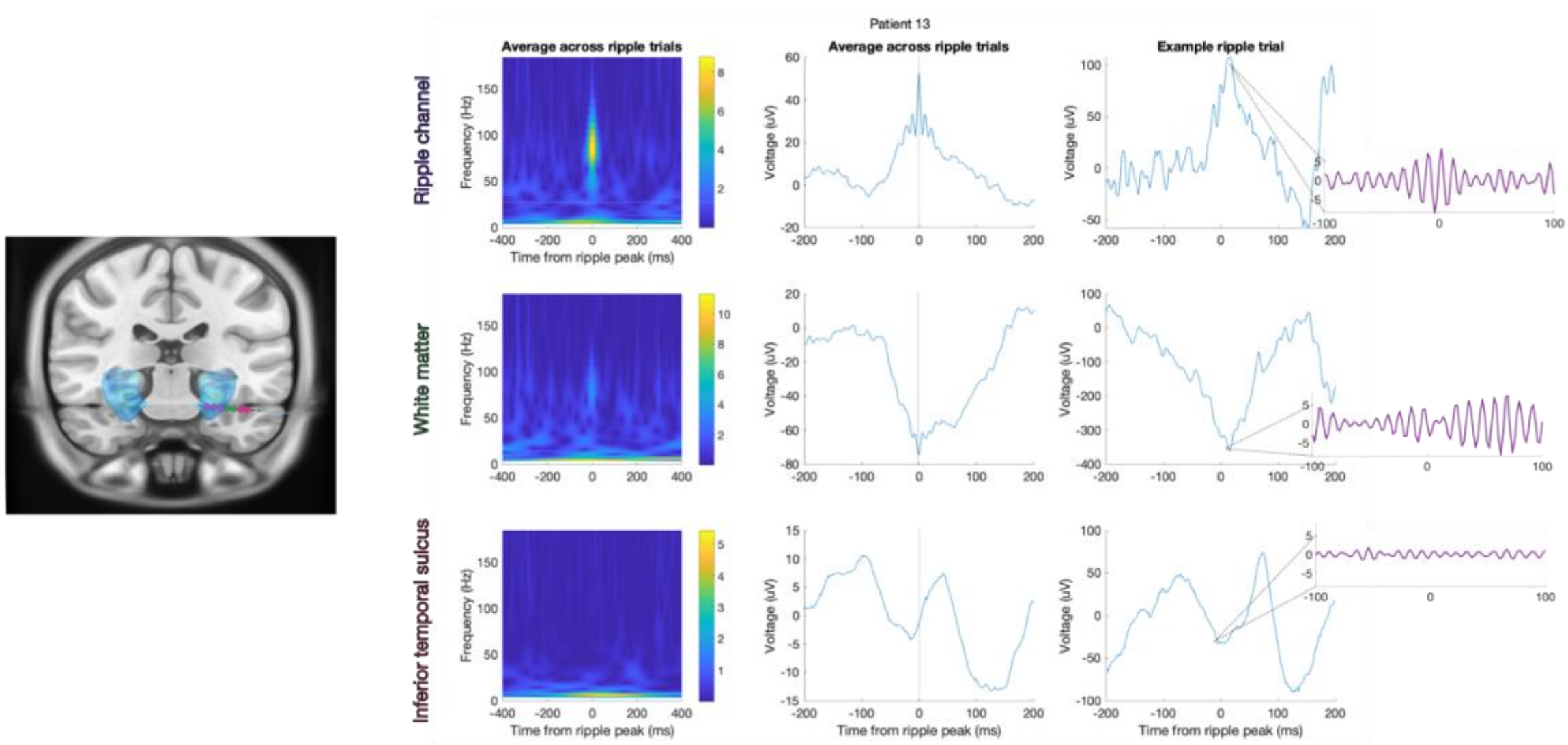

Figure S8 | Averaged, and example single trial, ripple traces from hippocampal and adjacent contacts for Patient 13. *Left*. Electrode localisation shown on the smoothed

hippocampus mask from the Automated Anatomical Labelling atlas for visualization; blue) illustrates the contacts (coloured circles) from which data are presented in the panels on the right. Purple circles: 3 hippocampal contacts i.e., two bipolar channels. Green circles: the two contacts adjacent to the hippocampus are in white matter. Red circles: the two subsequent contacts are in inferior temporal sulcus. *Right*. Top, bottom and middle rows: ripple-locked activity from the hippocampal (Ripple) channels (purple circles), and adjacent bipolar channels “White matter” (corresponding to green circles) and “inferior temporal sulcus” (corresponding to red circles). The left and centre columns show the average peri-ripple wavelet spectrogram across all ripple trials (n = 122), and average raw field potential centred on ripple peak, respectively. The right column shows an example ripple trial (raw field potential centred on ripple peak) with an inset showing the ripple band activity (band pass filtered at 80-120Hz).

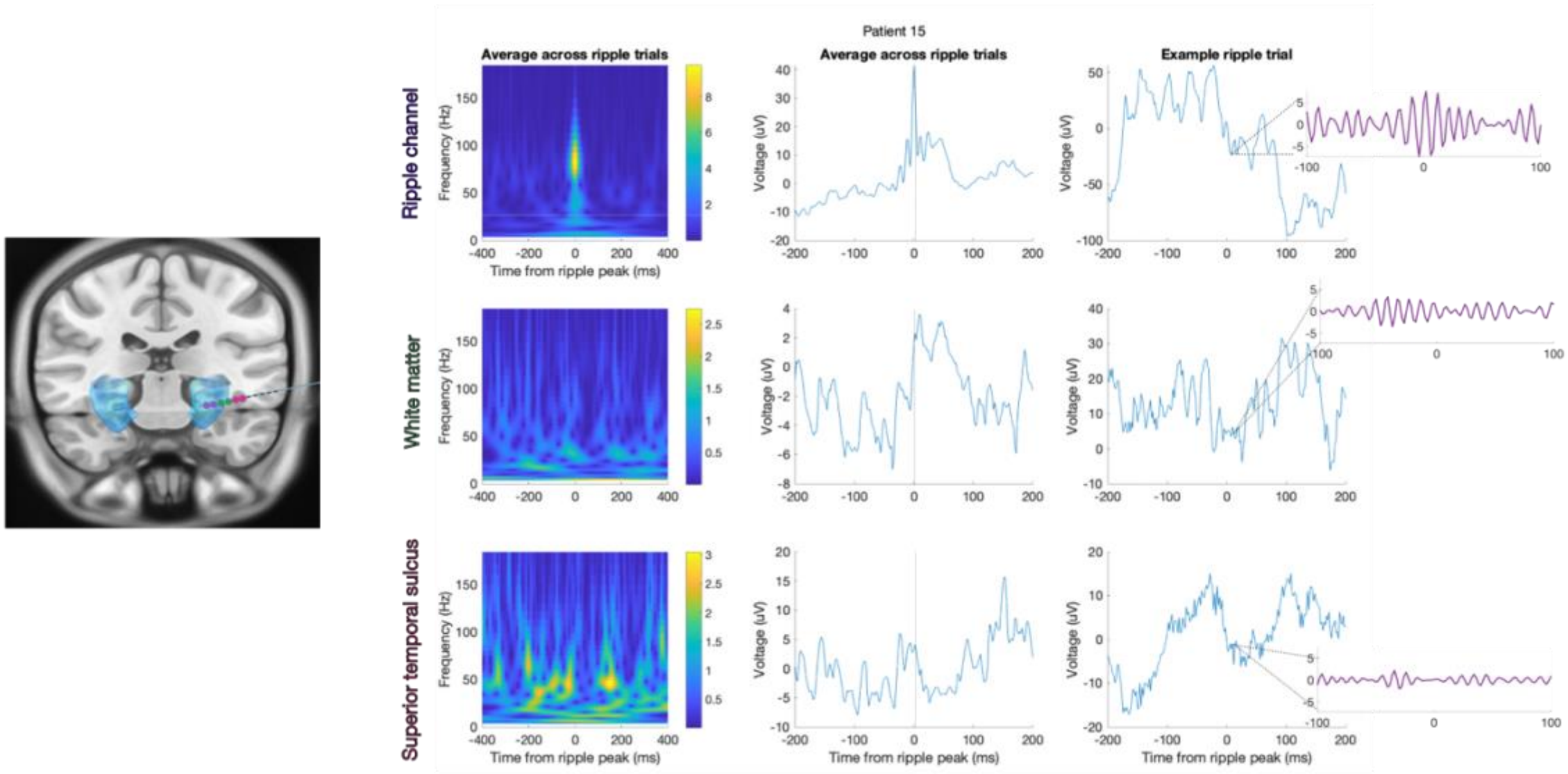

Figure S9 | Averaged, and example single trial, ripple traces from hippocampal and adjacent contacts for Patient 15. *Left*. Electrode localisation shown on the smoothed hippocampus mask from the Automated Anatomical Labelling atlas for visualization; blue) illustrates the contacts (coloured circles) from which data are presented in the panels on the right. Purple circles: 2 hippocampal contacts i.e., one bipolar channel. Green circles: the two contacts adjacent to the hippocampus are in white matter. Red circles: the two subsequent contacts are in superior temporal sulcus (medial contact)/white matter (lateral contact). *Right*. Top, bottom and middle rows: ripple-locked activity from the hippocampal (Ripple) channel (purple circles), and adjacent bipolar channels “White matter” (corresponding to green circles) and “Superior temporal sulcus” (corresponding to red circles). The left and centre columns show the average peri-ripple wavelet spectrogram across all ripple trials (n = 169), and average raw field potential centred on ripple peak, respectively. The right column shows an example ripple trial (raw field potential centred on ripple peak) with an inset showing the ripple band activity (band pass filtered at 80-120Hz).

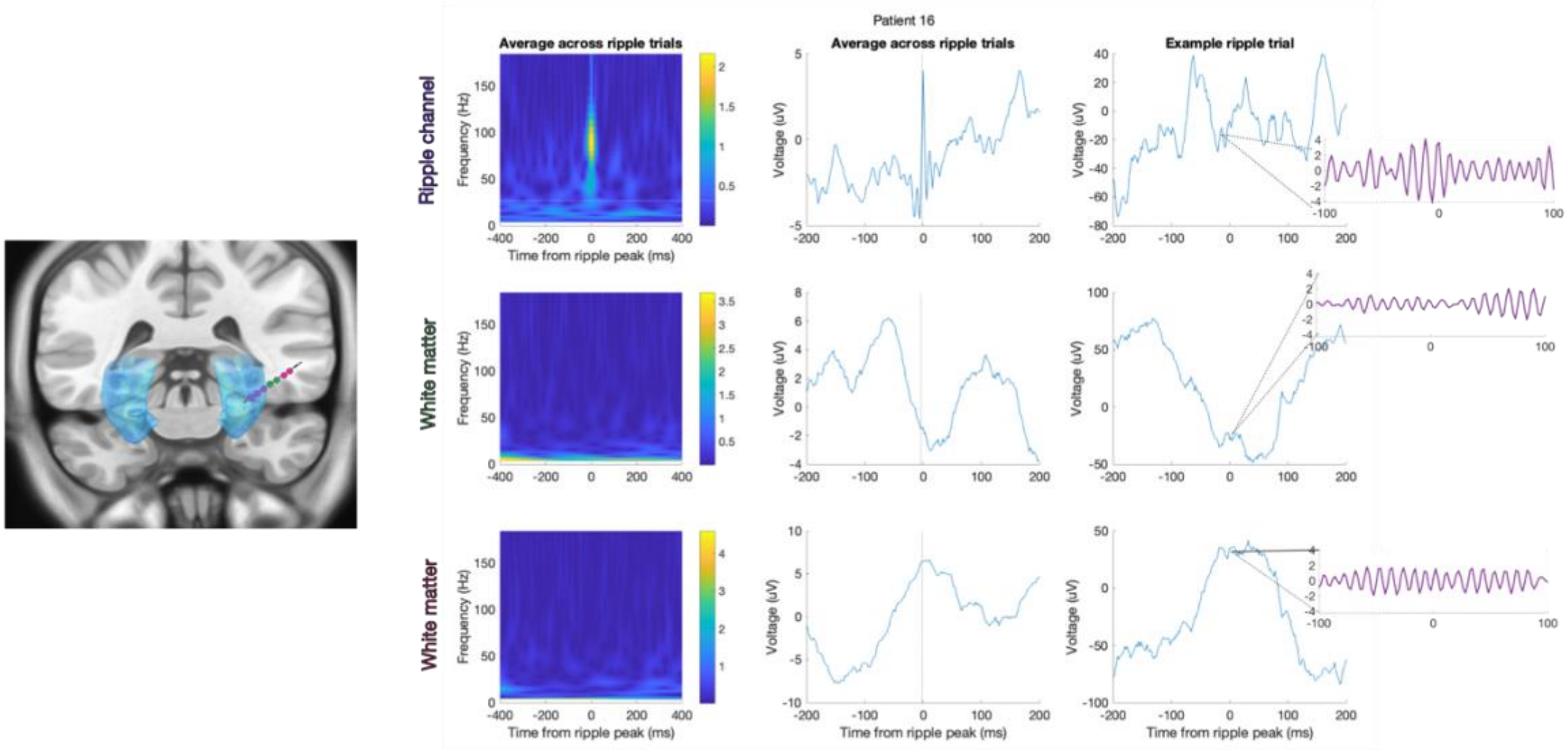

Figure S10 | Averaged, and example single trial, ripple traces from hippocampal and adjacent contacts for Patient 16. *Left*. Electrode localisation shown on the smoothed hippocampus mask from the Automated Anatomical Labelling atlas for visualization; blue) illustrates the contacts (coloured circles) from which data are presented in the panels on the right. Purple circles: 2 hippocampal contacts i.e., one bipolar channel. Green circles: the two contacts adjacent to the hippocampus are in white matter. Red circles: the two subsequent contacts are in white matter. *Right*. Top, bottom and middle rows: ripple-locked activity from the hippocampal (Ripple) channel (purple circles), and adjacent bipolar channels “White matter” (corresponding to green circles) and “White matter” (corresponding to red circles) . The left and centre columns show the average peri-ripple wavelet spectrogram across all ripple trials (n = 266), and average raw field potential centred on ripple peak, respectively. The right column shows an example ripple trial (raw field potential centred on ripple peak) with an inset showing the ripple band activity (band pass filtered at 80-120Hz).

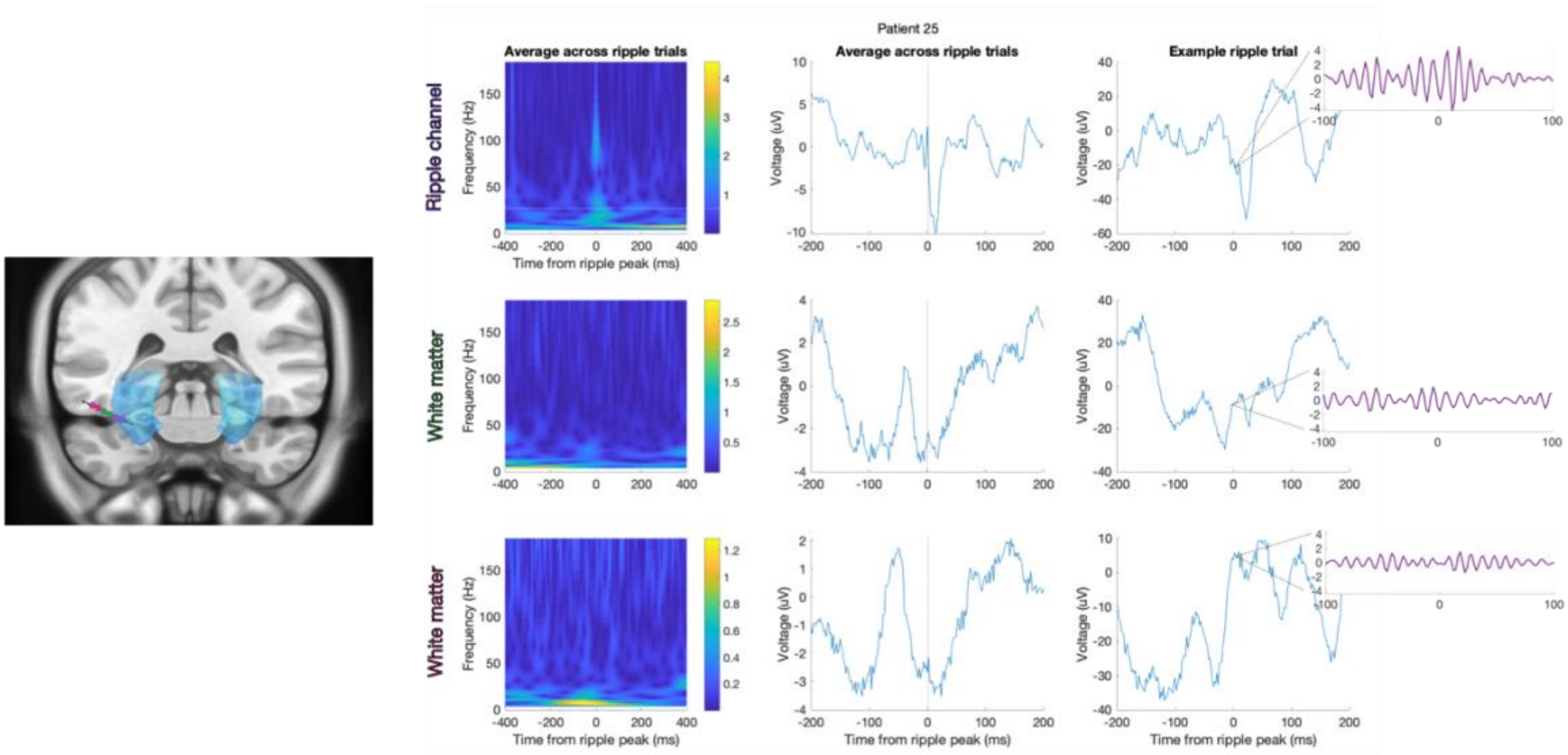

Figure S11 | Averaged, and example single trial, ripple traces from hippocampal and adjacent contacts for Patient 25. *Left*. Electrode localisation shown on the smoothed

hippocampus mask from the Automated Anatomical Labelling atlas for visualization; blue) illustrates the contacts (coloured circles) from which data are presented in the panels on the right. Purple circles: 2 hippocampal contacts i.e., one bipolar channel. Green circles: the two contacts adjacent to the hippocampus are in white matter. Red circles: the two subsequent contacts are in white matter. *Right*. Top, bottom and middle rows: ripple-locked activity from the hippocampal (Ripple) channels (purple circles), and adjacent bipolar channels "White matter" (corresponding to green circles) and "White matter" (corresponding to red circles) . The left and centre columns show the average peri-ripple wavelet spectrogram across all ripple trials (n = 49), and average raw field potential centred on ripple peak, respectively. The right column shows an example ripple trial (raw field potential centred on ripple peak) with an inset showing the ripple band activity (band pass filtered at 80-120Hz).

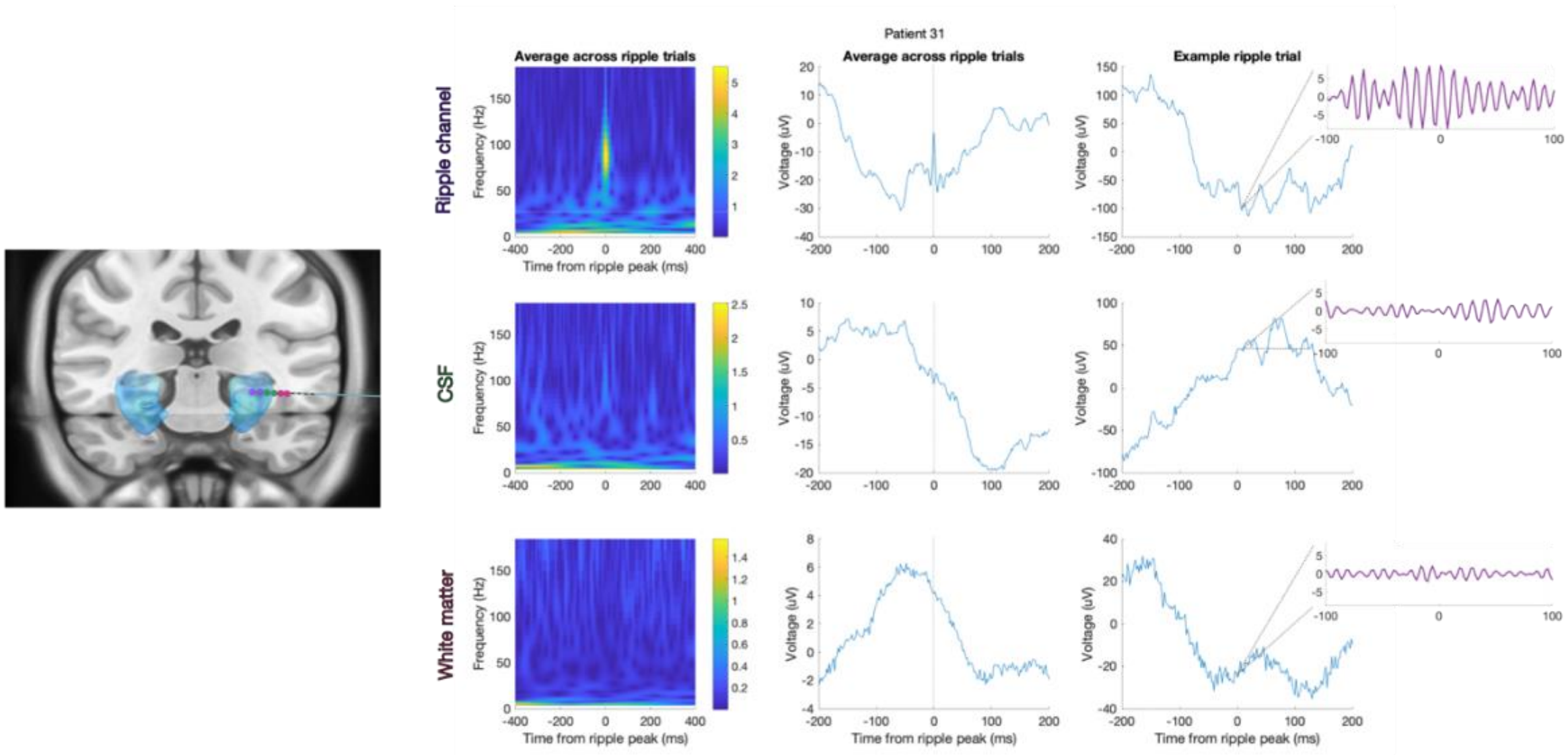

Figure S12 | Averaged, and example single trial, ripple traces from hippocampal and adjacent contacts for Patient 31. *Left*. Electrode localisation shown on the smoothed hippocampus mask from the Automated Anatomical Labelling atlas for visualization; blue) illustrates the contacts (coloured circles) from which data are presented in the panels on the right. Purple circles: 2 hippocampal contacts i.e., one bipolar channel. Green circles: the two contacts adjacent to the hippocampus are in CSF (medial contact)/white matter (lateral contact). Red circles: the two subsequent contacts are in white matter. *Right*. Top, bottom and middle rows: ripple-locked activity from the hippocampal (Ripple) channel (purple circles), and adjacent bipolar channels "CSF" (corresponding to green circles) and "White matter" (corresponding to red circles). The left and centre columns show the average peri-ripple wavelet spectrogram across all ripple trials (n = 100), and average raw field potential centred on ripple peak, respectively. The right column shows an example ripple trial (raw field potential centred on ripple peak) with an inset showing the ripple band activity (band pass filtered at 80-120Hz).

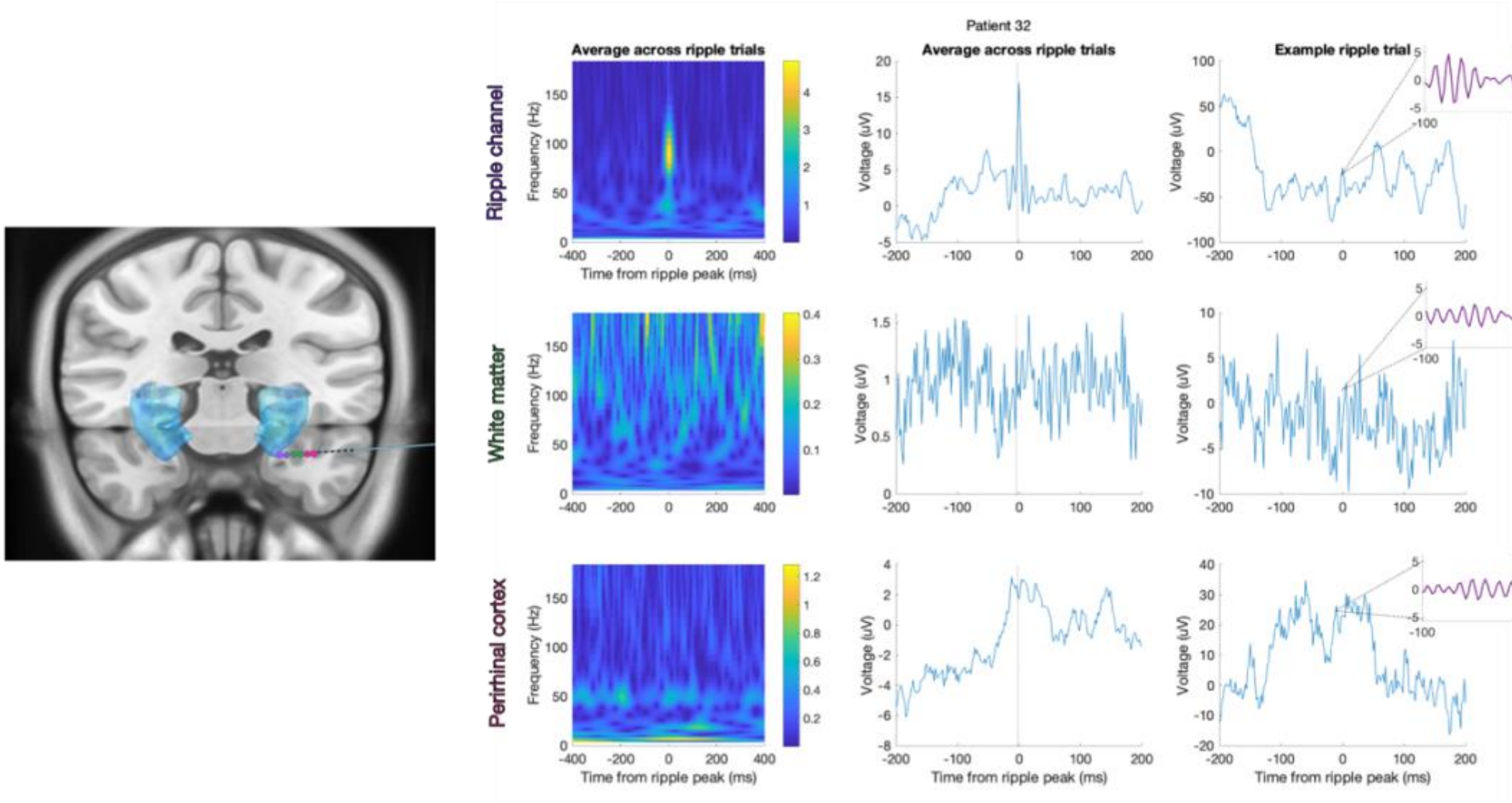

Figure S13 | Averaged, and example single trial, ripple traces from hippocampal and adjacent contacts for Patient 32. *Left*. Electrode localisation shown on the smoothed hippocampus mask from the Automated Anatomical Labelling atlas for visualization; blue) illustrates the contacts (coloured circles) from which data are presented in the panels on the right. Purple circles: 2 hippocampal contacts i.e., one bipolar channel. Green circles: the two contacts adjacent to the hippocampus are in white matter (medial contact)/CSF (lateral contact). Red circles: the two subsequent contacts are in perirhinal cortex. *Right*. Top, bottom and middle rows: ripple-locked activity from the hippocampal (Ripple) channel (purple circles), and adjacent bipolar channels "White matter" (corresponding to green circles) and "Perirhinal cortex" (corresponding to red circles). The left and centre columns show the average peri-ripple wavelet spectrogram across all ripple trials (n = 120), and average raw field potential centred on ripple peak, respectively. The right column shows an example ripple trial (raw field potential centred on ripple peak) with an inset showing the ripple band activity (band pass filtered at 80-120Hz).

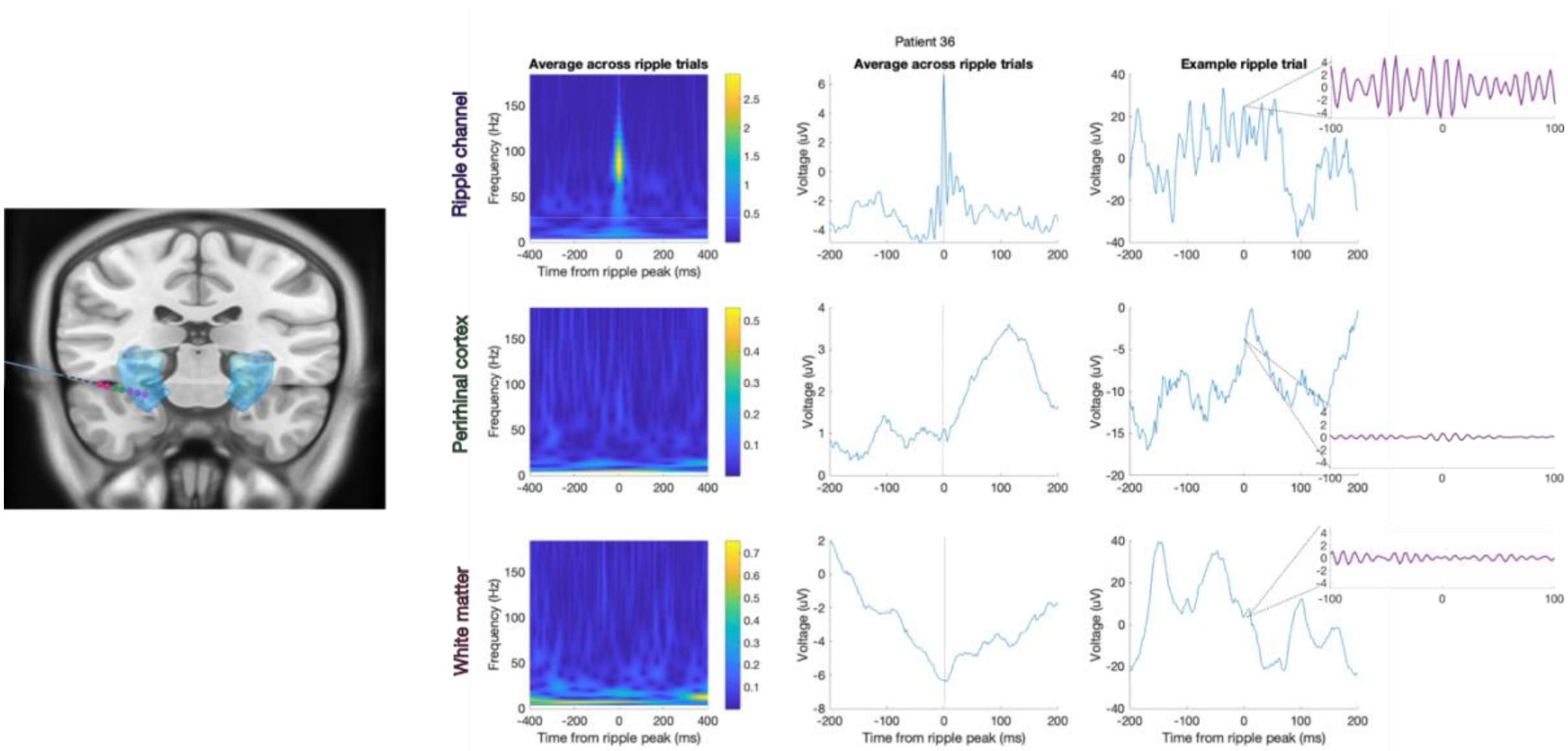

Figure S14 | Averaged, and example single trial, ripple traces from hippocampal and adjacent contacts for Patient 36. *Left*. Electrode localisation shown on the smoothed hippocampus mask from the Automated Anatomical Labelling atlas for visualization; blue)

illustrates the contacts (coloured circles) from which data are presented in the panels on the right. Purple circles: 3 hippocampal contacts i.e., two bipolar channels. Green circles: the two contacts adjacent to the hippocampus are in perirhinal cortex. Red circles: the two subsequent contacts are in white matter (medial contact)/CSF (lateral contact). *Right*. Top, bottom and middle rows: ripple-locked activity from the hippocampal (Ripple) channels (purple circles), and adjacent bipolar channels "Perirhinal cortex" (corresponding to green circles) and "White matter" (corresponding to red circles). The left and centre columns show the average peri-ripple wavelet spectrogram across all ripple trials (n = 381), and average raw field potential centred on ripple peak, respectively. The right column shows an example ripple trial (raw field potential centred on ripple peak) with an inset showing the ripple band activity (band pass filtered at 80-120Hz).

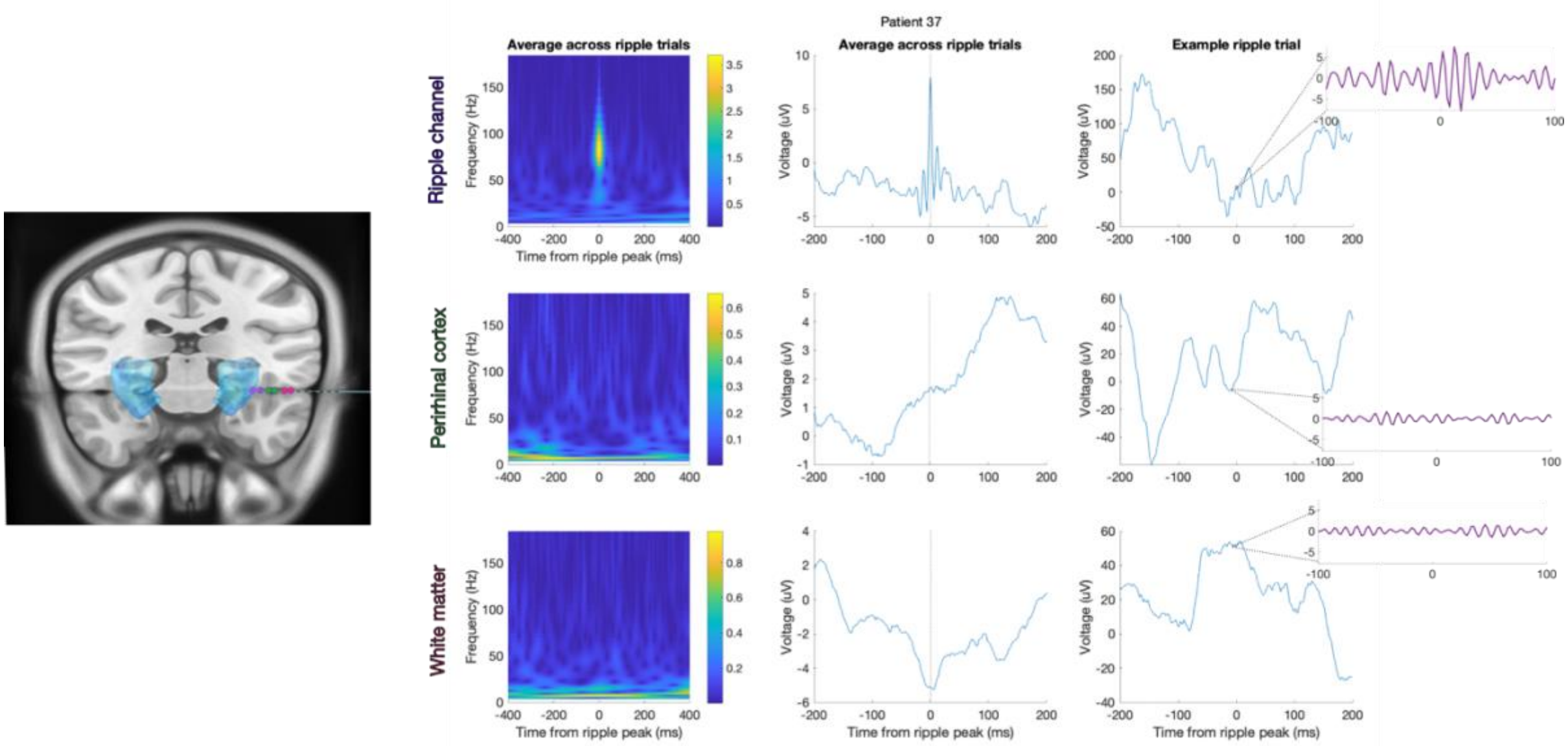

Figure S15 | Averaged, and example single trial, ripple traces from hippocampal and adjacent contacts for Patient 37. *Left*. Electrode localisation shown on the smoothed hippocampus mask from the Automated Anatomical Labelling atlas for visualization; blue) illustrates the contacts (coloured circles) from which data are presented in the panels on the right. Purple circles: 2 hippocampal contacts i.e., one bipolar channel. Green circles: the two contacts adjacent to the hippocampus are in perirhinal cortex (medial contact)/white matter (lateral contact). Red circles: the two subsequent contacts are in white matter (medial contact)/inferior temporal sulcus (lateral contact). *Right*. Top, bottom and middle rows: ripple-locked activity from the hippocampal (Ripple) channel (purple circles), and adjacent bipolar channels "Perirhinal cortex" (corresponding to green circles) and "White matter" (corresponding to red circles). The left and centre columns show the average peri-ripple wavelet spectrogram across all ripple trials (n = 80), and average raw field potential centred on ripple peak, respectively. The right column shows an example ripple trial (raw field potential centred on ripple peak) with an inset showing the ripple band activity (band pass filtered at 80-120Hz).

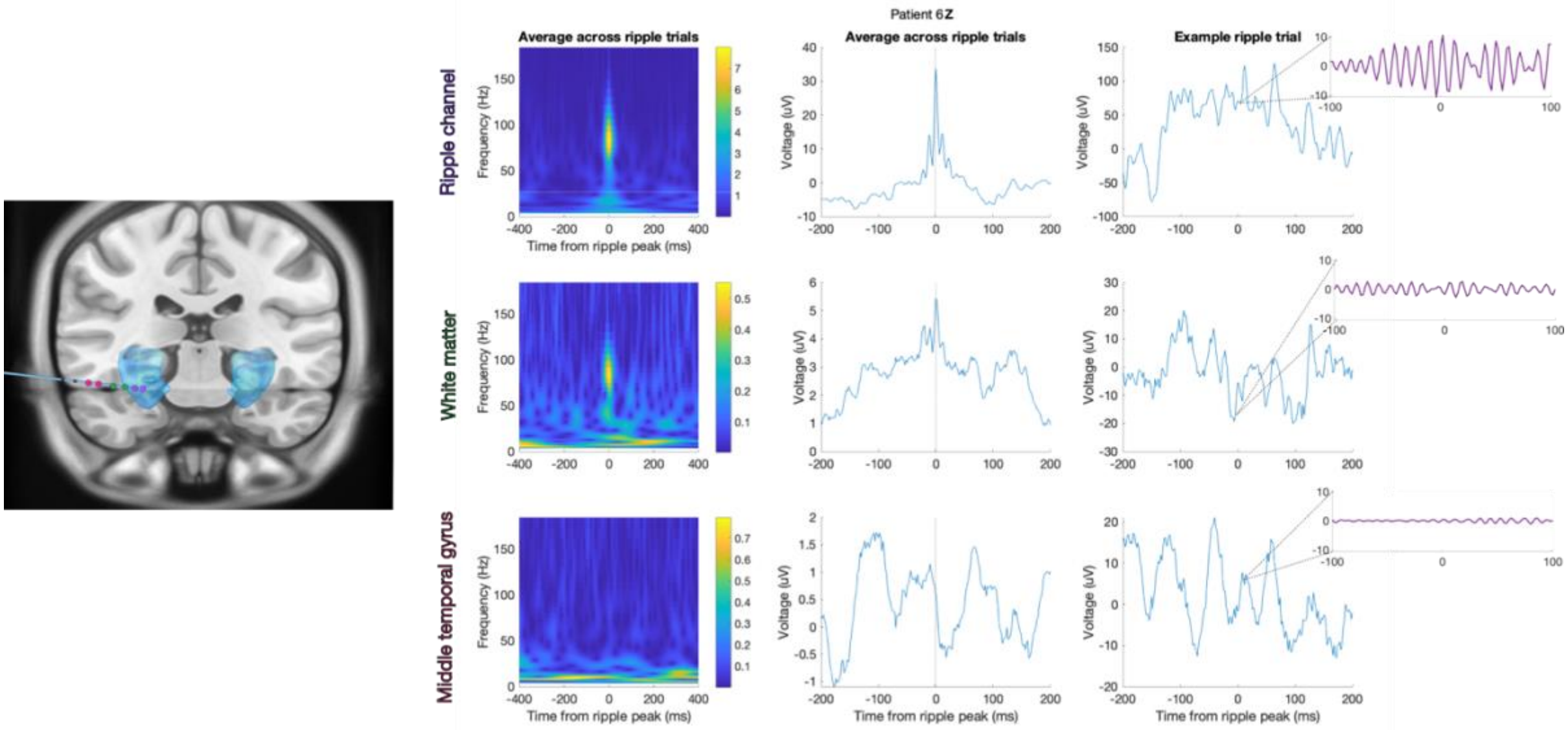

Figure S16 | Averaged, and example single trial, ripple traces from hippocampal and adjacent contacts for Patient 6z. *Left*. Electrode localisation shown on the smoothed hippocampus mask from the Automated Anatomical Labelling atlas for visualization; blue) illustrates the contacts (coloured circles) from which data are presented in the panels on the right. Purple circles: 2 hippocampal contacts i.e., one bipolar channel. Green circles: the two contacts adjacent to the hippocampus are in white matter. Red circles: the two subsequent contacts are in middle temporal gyrus. *Right*. Top, bottom and middle rows: ripple-locked activity from the hippocampal (Ripple) channel (purple circles), and adjacent bipolar channels “White matter” (corresponding to green circles) and “Middle temporal gyrus” (corresponding to red circles). The left and centre columns show the average peri-ripple wavelet spectrogram across all ripple trials (n = 253), and average raw field potential centred on ripple peak, respectively. The right column shows an example ripple trial (raw field potential centred on ripple peak) with an inset showing the ripple band activity (band pass filtered at 80-120Hz).

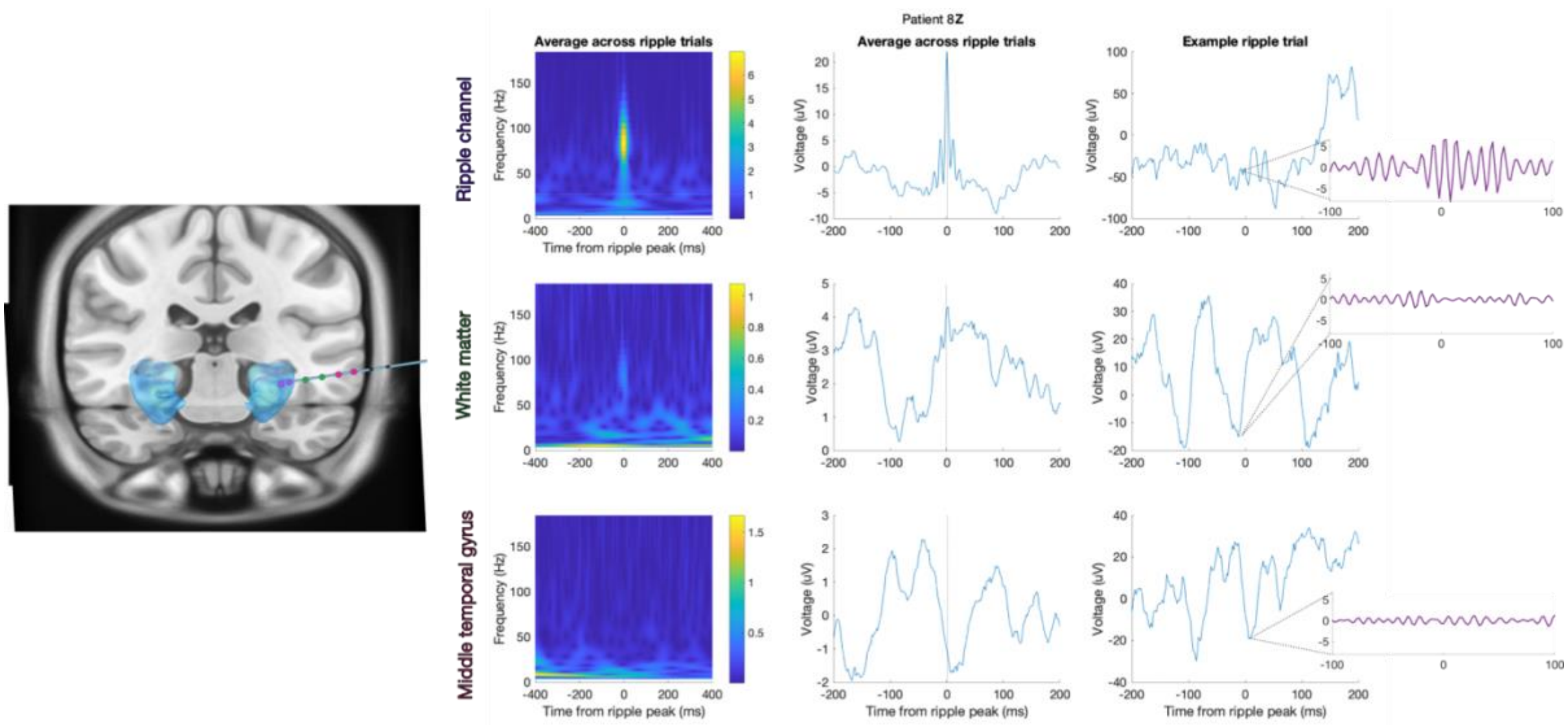

Figure S17 | Averaged, and example single trial, ripple traces from hippocampal and adjacent contacts for Patient 8z. *Left*. Electrode localisation shown on the smoothed hippocampus mask from the Automated Anatomical Labelling atlas for visualization; blue) illustrates the contacts (coloured circles) from which data are presented in the panels on the right. Purple circles: 2 hippocampal contacts i.e., one bipolar channel. Green circles: the two contacts adjacent to the hippocampus are in white matter. Red circles: the two subsequent

contacts are in middle temporal gyrus (medial contact)/white matter (lateral contact). *Right*. Top, bottom and middle rows: ripple-locked activity from the hippocampal (Ripple) channel (purple circles), and adjacent bipolar channels "White matter" (corresponding to green circles) and "Middle temporal gyrus" (corresponding to red circles). The left and centre columns show the average peri-ripple wavelet spectrogram across all ripple trials (n = 92), and average raw field potential centred on ripple peak, respectively. The right column shows an example ripple trial (raw field potential centred on ripple peak) with an inset showing the ripple band activity (band pass filtered at 80-120Hz).

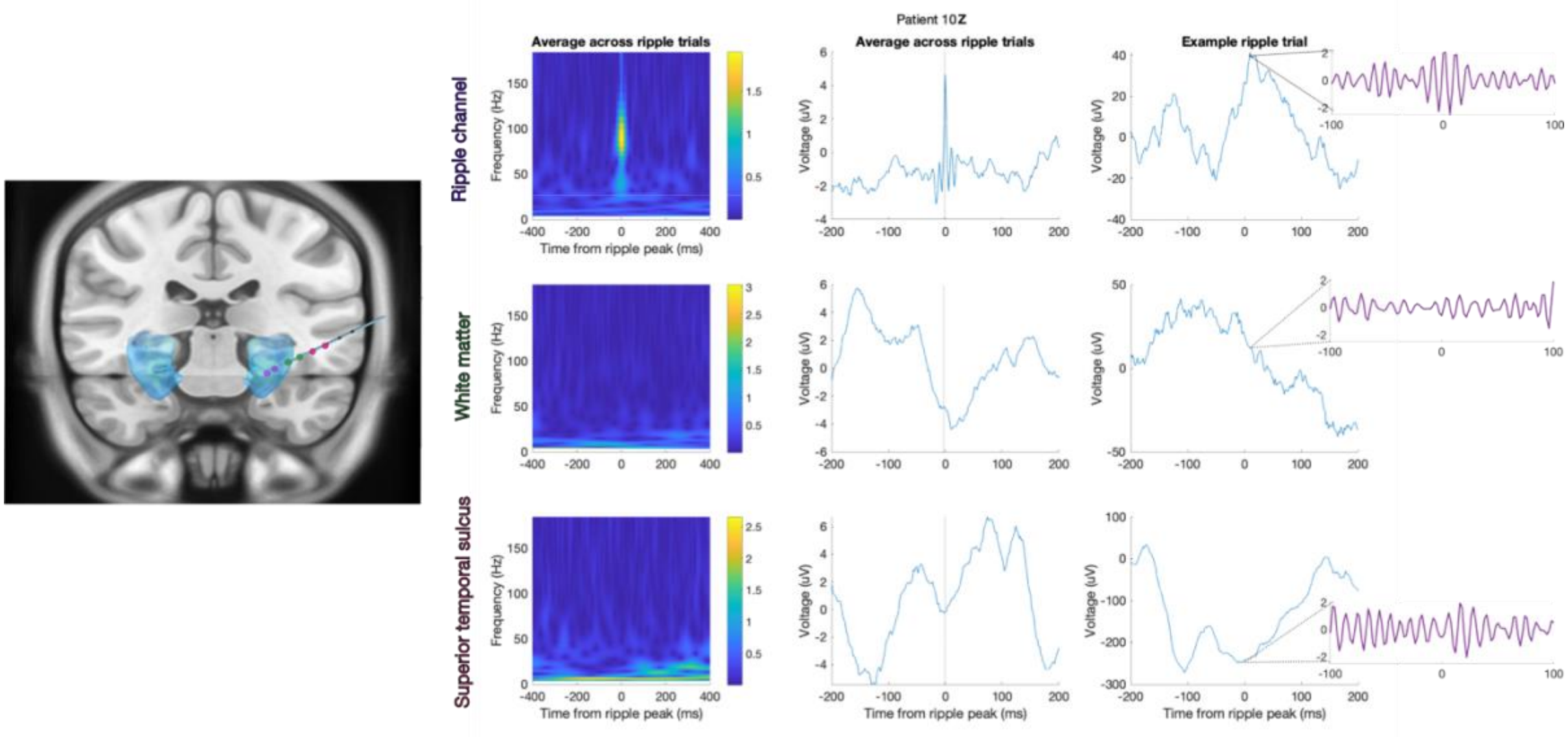

Figure S18 | Averaged, and example single trial, ripple traces from hippocampal and adjacent contacts for Patient 10z. *Left*. Electrode localisation shown on the smoothed hippocampus mask from the Automated Anatomical Labelling atlas for visualization; blue) illustrates the contacts (coloured circles) from which data are presented in the panels on the right. Purple circles: 2 hippocampal contacts i.e., one bipolar channel. Green circles: the two contacts adjacent to the hippocampus are in white matter. Red circles: the two subsequent contacts are in superior temporal sulcus. *Right*. Top, bottom and middle rows: ripple-locked activity from the hippocampal (Ripple) channel (purple circles), and adjacent bipolar channels "White matter" (corresponding to green circles) and "Superior temporal sulcus" (corresponding to red circles). The left and centre columns show the average peri-ripple wavelet spectrogram across all ripple trials (n = 120), and average raw field potential centred on ripple peak, respectively. The right column shows an example ripple trial (raw field potential centred on ripple peak) with an inset showing the ripple band activity (band pass filtered at 80-120Hz).

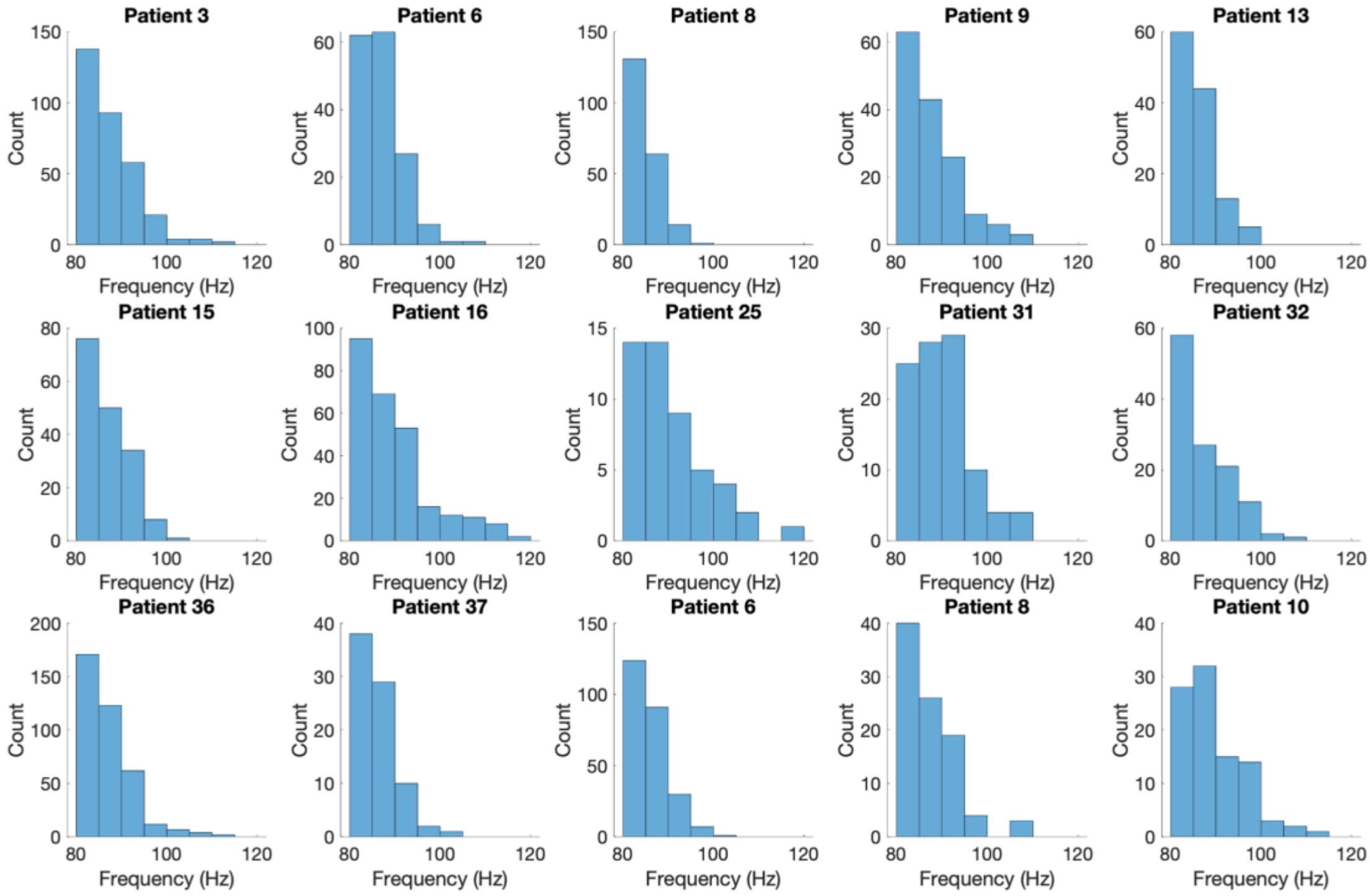

Figure S19 | Ripple peak frequency per patient. Histograms showing the distribution of each patient's ripples peak frequency. The majority of ripples occur at a frequency between 80 and 90 Hz (average ripple frequency over patients 87.5 Hz standard deviation 5.9).

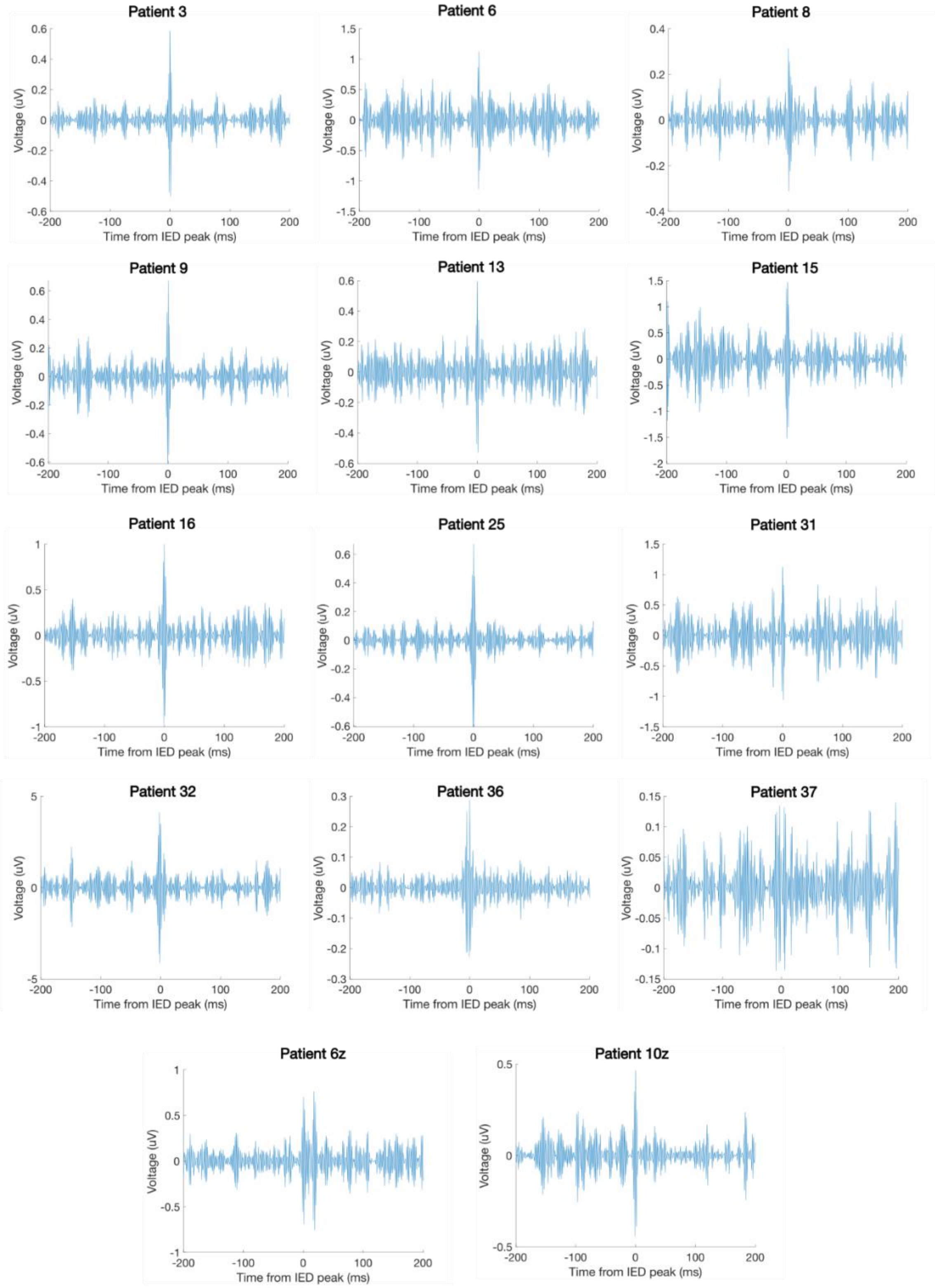

Figure S20 | Mean IEDs per patient. High-pass filtered LFP (200Hz) across all detected IEDs, per patient.

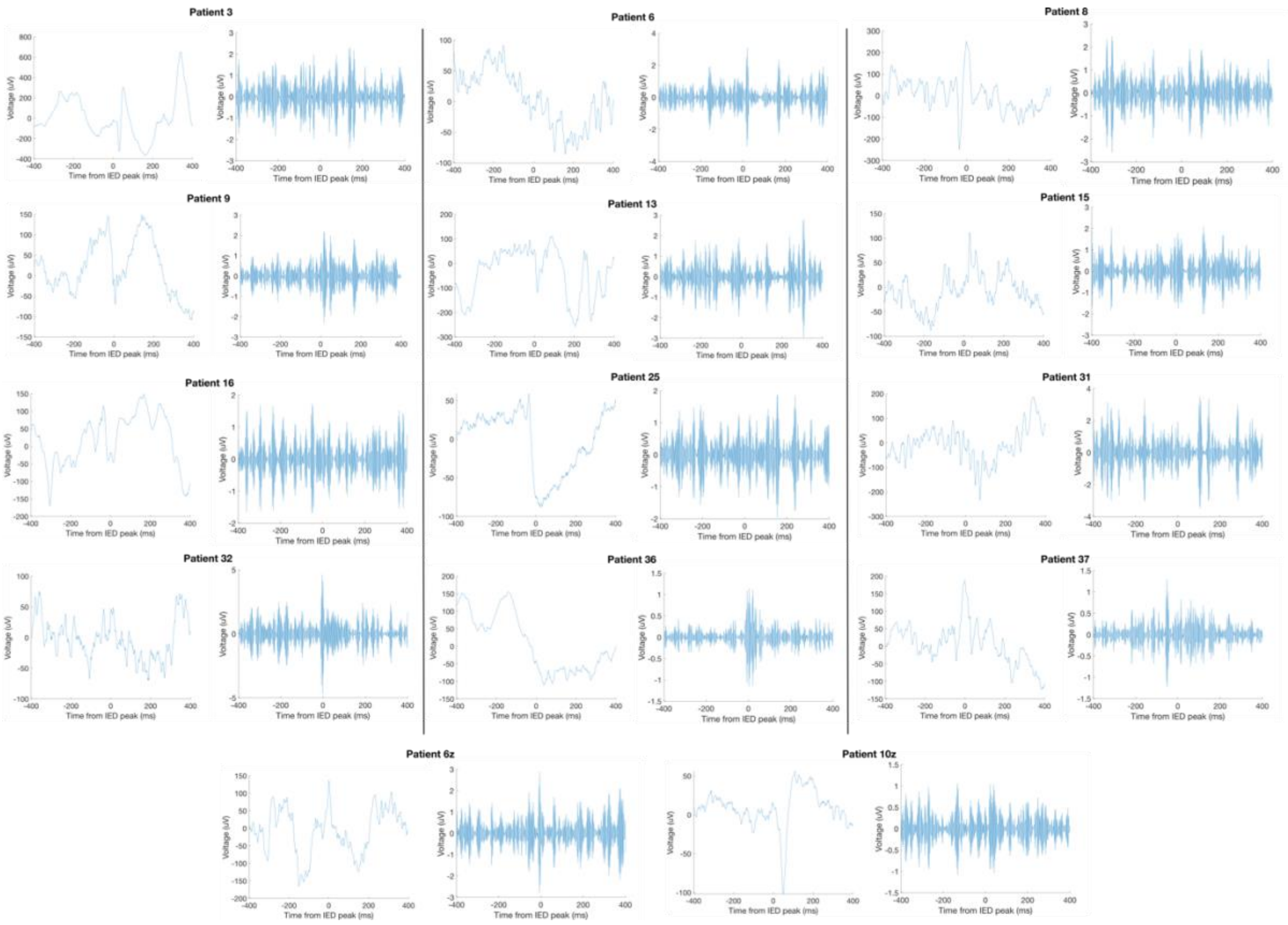

Figure S21 | Example IEDs per patient. Raw and high-pass filtered LFP (200Hz) for an example IED, per patient.
