# Supplementary material for: Human hippocampal ripples tune cortical responses based on predicted uncertainty
Source: Nat Neurosci. 2026 Jun 25;29(8):1987–98. doi: 10.1038/s41593-026-02345-6 (PMC13433285; doi:10.1038/s41593-026-02345-6)
Supplement: Supplementary file 2 [file 41593_2026_2345_MOESM2_ESM.pdf]

nature portfolio

Corresponding author(s): Darya Frank
Bryan Strange

Last updated by author(s): 30/04/2026

Reporting Summary

Nature Portfolio wishes to improve the reproducibility of the work that we publish. This form provides structure for consistency and transparency in reporting. For further information on Nature Portfolio policies, see our Editorial Policies and the Editorial Policy Checklist.

Statistics

For all statistical analyses, confirm that the following items are present in the figure legend, table legend, main text, or Methods section.

n/a	Confirmed	
☐	☒	The exact sample size (n) for each experimental group/condition, given as a discrete number and unit of measurement
☐	☒	A statement on whether measurements were taken from distinct samples or whether the same sample was measured repeatedly
☐	☒	The statistical test(s) used AND whether they are one- or two-sided<br>*Only common tests should be described solely by name; describe more complex techniques in the Methods section.*
☐	☒	A description of all covariates tested
☐	☒	A description of any assumptions or corrections, such as tests of normality and adjustment for multiple comparisons
☐	☒	A full description of the statistical parameters including central tendency (e.g. means) or other basic estimates (e.g. regression coefficient) AND variation (e.g. standard deviation) or associated estimates of uncertainty (e.g. confidence intervals)
☐	☒	For null hypothesis testing, the test statistic (e.g. F, t, r) with confidence intervals, effect sizes, degrees of freedom and P value noted<br>*Give P values as exact values whenever suitable.*
☒	☐	For Bayesian analysis, information on the choice of priors and Markov chain Monte Carlo settings
☒	☐	For hierarchical and complex designs, identification of the appropriate level for tests and full reporting of outcomes
☐	☒	Estimates of effect sizes (e.g. Cohen's d, Pearson's r), indicating how they were calculated

Our web collection on statistics for biologists contains articles on many of the points above.

Software and code

Policy information about availability of computer code

Data collection	Data was collected in two locations: in Madrid ongoing intracranial EEG activity was acquired using XLTEK EMU128FS amplifier (XLTEK, Ontario, Canada). In Zurich, intracranial recordings were acquired using a Neuralynx ATLAS system.
Data analysis	Data was analysed in Matlab 2019b using the Fieldtrip toolbox (version 20200310) and custom-code available in the following Github repository: https://github.com/frdarya/GenerativeRipples

For manuscripts utilizing custom algorithms or software that are central to the research but not yet described in published literature, software must be made available to editors and reviewers. We strongly encourage code deposition in a community repository (e.g. GitHub). See the Nature Portfolio guidelines for submitting code & software for further information.

Data

Policy information about availability of data

All manuscripts must include a data availability statement. This statement should provide the following information, where applicable:

- Accession codes, unique identifiers, or web links for publicly available datasets
- A description of any restrictions on data availability
- For clinical datasets or third party data, please ensure that the statement adheres to our policy

All data needed to generate the figures, and over which statistics were computed, are available in the following Github repository: https://github.com/frdarya/GenerativeRipples.

Human research participants

Policy information about studies involving human research participants and Sex and Gender in Research.

Reporting on sex and gender	Of the 18 patients who participated (17 who fulfill the inclusion criteria) 8 were males.
Population characteristics	Eighteen medication-resistant presurgical epilepsy patients (8 males; age range 24-59, mean age = 37.3 SD = 11.7), see Table S1 for full details
Recruitment	We included in the study all patients that agreed to participate and that had electrodes implantation in the hippocampus. Implantation sites were chosen solely on the basis of clinical criteria.
Ethics oversight	All patients signed informed consent. The study had full approval from the local ethics committees of the Hospital Ruber Internacional, Madrid, Spain and Kantonale Ethikkommission, Zurich, Switzerland (PB-2016-02055).

Note that full information on the approval of the study protocol must also be provided in the manuscript.

Field-specific reporting

Please select the one below that is the best fit for your research. If you are not sure, read the appropriate sections before making your selection.

☒ Life sciences ☐ Behavioural & social sciences ☐ Ecological, evolutionary & environmental sciences

For a reference copy of the document with all sections, see nature.com/documents/nr-reporting-summary-flat.pdf

Life sciences study design

All studies must disclose on these points even when the disclosure is negative.

Sample size	No statistical methods were used to pre-determine sample sizes but our sample sizes are larger or equal to those reported in previous publications (Zheng et al, 2017; Mendez-Bertolo et al 2016; Costa et al., 2022).
Data exclusions	We included in the analyses all patients that agreed to participate and completed the task (at least 25% of trials after exclusion due to performance or electrophysiological artefacts) and that had electrodes implantation in the hippocampus. Implantation sites were chosen solely on the basis of clinical criteria.
Replication	No out-of-sample replications were attempted, single-subject datapoints are presented in main and supplementary figures to demonstrate within-study between-subjects effects are consistent.
Randomization	The design is a within-subject with trial-by-trial variation (no categorical conditions), the underlying probability distribution of stimuli was randomly chosen per patient prior to the task from all possible combinations.
Blinding	Patients were not allocated into different experimental groups, therefore blinding was not relevant for data collection. Participants were naive to the task goals.

Reporting for specific materials, systems and methods

We require information from authors about some types of materials, experimental systems and methods used in many studies. Here, indicate whether each material, system or method listed is relevant to your study. If you are not sure if a list item applies to your research, read the appropriate section before selecting a response.

Materials & experimental systems

n/a	Involved in the study	
☒	☐	Antibodies
☒	☐	Eukaryotic cell lines
☒	☐	Palaeontology and archaeology
☒	☐	Animals and other organisms
☒	☐	Clinical data
☒	☐	Dual use research of concern

Methods

n/a	Involved in the study	
☒	☐	ChIP-seq
☒	☐	Flow cytometry
☐	☒	MRI-based neuroimaging

Magnetic resonance imaging

Experimental design

Design type	Clinical protocol for structural scanning
Design specifications	Structural scans used for electrode contact localization.
Behavioral performance measures	N/A

Acquisition

Imaging type(s)	Structural
Field strength	3
Sequence & imaging parameters	T1
Area of acquisition	Whole-brain

Diffusion MRI ☐ Used ☒ Not used

Preprocessing

Preprocessing software	SPM8 (http://www.fil.ion.ucl.ac.uk/spm).
Normalization	Data were normalized to MNI space
Normalization template	MNI
Noise and artifact removal	N/A
Volume censoring	N/A

Statistical modeling & inference

Model type and settings	N/A
Effect(s) tested	N/A

Specify type of analysis: ☐ Whole brain ☐ ROI-based ☐ Both

Statistic type for inference (See Eklund et al. 2016)	N/A
Correction	N/A

Models & analysis

n/a	Involved in the study
☒	☐ Functional and/or effective connectivity
☒	☐ Graph analysis
☒	☐ Multivariate modeling or predictive analysis
