# Supplementary material for: Human hippocampal ripples tune cortical responses based on predicted uncertainty
Source: Nat Neurosci. 2026 Jun 25;29(8):1987–98. doi: 10.1038/s41593-026-02345-6 (PMC13433285; doi:10.1038/s41593-026-02345-6)
Supplement: Supplementary file 3 [file 41593_2026_2345_MOESM3_ESM.pdf]

nature portfolio

Peer Review File

Human hippocampal ripples tune cortical responses based on predicted uncertainty

Corresponding Author: Dr Darya Frank

This manuscript has been previously reviewed at another journal. This document only contains information relating to versions considered at Nature Neuroscience.

Version 0:

Reviewer comments:

Reviewer #1

(Comments for the Author)
This manuscript is a revision of a prior submission that was previously reviewed for Nature. Based on the initial round of reviews, the authors were advised to submit a revised version to Nature Neuroscience. I have read over the revised manuscript, and the response of the authors to my previous comments in the initial review. The study is certainly interesting and the conclusions and data are valuable, especially in positioning the role of ripples in the predictive coding framework. In that sene, it would be appropriate for Nature Neuroscience.

Regarding the specific comments raised in the review, I commend the authors for providing a full and comprehensive revision to the original manuscript. I do not have access to the other reviews, but it is clear that the authors have sufficiently addressed the comments that I raised in the initial review. The statistical support for the results are more robust and the authors provide converging evidence for their conclusions.

Open Access This Peer Review File is licensed under a Creative Commons Attribution 4.0 International License, which permits use, sharing, adaptation, distribution and reproduction in any medium or format, as long as you give appropriate credit to the original author(s) and the source, provide a link to the Creative Commons license, and indicate if changes were made.
In cases where reviewers are anonymous, credit should be given to 'Anonymous Referee' and the source.
The images or other third party material in this Peer Review File are included in the article's Creative Commons license, unless indicated otherwise in a credit line to the material. If material is not included in the article's Creative Commons license and your intended use is not permitted by statutory regulation or exceeds the permitted use, you will need to obtain permission directly from the copyright holder.
To view a copy of this license, visit https://creativecommons.org/licenses/by/4.0/
